# JMJD6 Dysfunction Due to Iron Deficiency in Preeclampsia Disrupts Fibronectin Homeostasis Resulting in Diminished Trophoblast Migration

**DOI:** 10.3389/fcell.2021.652607

**Published:** 2021-05-12

**Authors:** Sruthi Alahari, Abby Farrell, Leonardo Ermini, Chanho Park, Julien Sallais, Sarah Roberts, Taylor Gillmore, Michael Litvack, Martin Post, Isabella Caniggia

**Affiliations:** ^1^Lunenfeld-Tanenbaum Research Institute, Sinai Health System, Toronto, ON, Canada; ^2^Department of Physiology, University of Toronto, Toronto, ON, Canada; ^3^Institute of Medical Science, University of Toronto, Toronto, ON, Canada; ^4^Program in Translational Medicine, Peter Gilgan Centre for Research and Learning, Hospital for Sick Children, Toronto, ON, Canada; ^5^Department of Obstetrics and Gynecology, University of Toronto, Toronto, ON, Canada

**Keywords:** fibronectin, iron, JMJD6, pMSCs, preeclampsia, trophoblast migration

## Abstract

The mechanisms contributing to excessive fibronectin in preeclampsia, a pregnancy-related disorder, remain unknown. Herein, we investigated the role of JMJD6, an O_2_- and Fe^2+^-dependent enzyme, in mediating placental fibronectin processing and function. MALDI-TOF identified fibronectin as a novel target of JMJD6-mediated lysyl hydroxylation, preceding fibronectin glycosylation, deposition, and degradation. In preeclamptic placentae, fibronectin accumulated primarily in lysosomes of the mesenchyme. Using primary placental mesenchymal cells (pMSCs), we found that fibronectin fibril formation and turnover were markedly impeded in preeclamptic pMSCs, partly due to impaired lysosomal degradation. JMJD6 knockdown in control pMSCs recapitulated the preeclamptic FN phenotype. Importantly, preeclamptic pMSCs had less total and labile Fe^2+^ and Hinokitiol treatment rescued fibronectin assembly and promoted lysosomal degradation. Time-lapse imaging demonstrated that defective ECM deposition by preeclamptic pMSCs impeded HTR-8/SVneo cell migration, which was rescued upon Hinokitiol exposure. Our findings reveal new Fe^2+^-dependent mechanisms controlling fibronectin homeostasis/function in the placenta that go awry in preeclampsia.

## Introduction

The glycoprotein fibronectin (FN) is a fundamental component of the extracellular matrix (ECM), actively mediating a variety of cellular events including differentiation, migration, invasion, and wound healing (Wierzbicka-Patynowski and Schwarzbauer, 2003). In humans, FN is ubiquitously expressed and exists in two forms, plasma and cellular; both forms vary in their chemical and biological properties, elicit distinct functions (Pankov and Yamada, 2002), and contribute to the formation of an insoluble fibrillar matrix that sustains the ECM.

While the precise mechanisms that orchestrate FN matrix assembly are yet to be elucidated, pioneering work has shown that FN fibrillogenesis is a cell-mediated affair. Soluble FN, produced in the endoplasmic reticulum (ER), is initially synthesized as monomers that are post-translationally modified and dimerize as they traffic to the Golgi apparatus. In this cellular compartment, dimers are folded into a compact conformation and secreted into the extracellular space (Johnson et al., 1999), where they serve as ligands for the FN high-affinity transmembrane receptor, α5β1 integrin. This interaction initiates a complex signaling cascade whereby short FN fibrils are eventually transformed into a dense, insoluble fibrillar network, forming functional links to other ECM proteins (Wierzbicka-Patynowski and Schwarzbauer, 2003).

In the placenta, FN exhibits diverse biological roles throughout gestation and is abundant in the basement membrane, mesenchymal stromal cells of the chorionic villi, fetal blood vessels, and in a subset of extravillous trophoblasts (EVTs) (Yamaguchi et al., 1985). The expression of FN and its receptor (α5β1 integrin) in EVTs is spatially and temporally regulated and orchestrates trophoblast differentiation toward a migratory/invasive phenotype (Damsky et al., 1992; Bischof et al., 1993; Caniggia et al., 1999). Interestingly, ECM derived from placental mesenchymal stromal cells (pMSCs) has been reported to trigger EVT migration and invasion *in vitro* through paracrine interactions (Chen and Aplin, 2003; Chen, 2014). On the other hand, FN signaling *via* α5β1 integrins mediates the differentiation of pMSCs into endothelial cells, further underscoring the critical role of FN in placental development (Lee et al., 2009). Despite its numerous functions, our current understanding of how placental FN is processed and assembled is limited.

The early placenta develops in a relatively hypoxic environment that is necessary for proper fetal growth (Rodesch et al., 1992). Hypoxia is among the most potent stimuli impacting on ECM structure and function (Gilkes et al., 2014), and in the developing human placenta, hypoxia upregulates FN transcript and protein levels (Genbacev et al., 2001; Chen and Aplin, 2003). Exposure of placental villous explants to low oxygen is accompanied by enhanced FN production and increased EVT outgrowth from distal villous tips (Caniggia et al., 1999). In contrast, chronic placental hypoxia, accompanied by excessive placental and plasma FN, has been implicated in the genesis of preeclampsia (PE), a devastating disorder of pregnancy linked to shallow trophoblast migration and invasion, and impaired remodeling of maternal spiral arteries (Brubaker et al., 1992; Fisher, 2015). Work from our laboratory and others has shown that in PE, EVTs retain high expression of FN and α5 integrin and fail to acquire an invasive phenotype (Damsky et al., 1994; Caniggia et al., 1999). Although placental hypoxia is central to the pathogenesis of PE, it is unclear whether aberrant FN homeostasis in this disease stems from altered placental oxygen sensing.

Recent studies on the epigenetic control of genes induced by hypoxia have highlighted the role of a family of dioxygenase enzymes termed Jumonji C (JmjC) domain containing proteins that utilize molecular oxygen (O_2_) and ferrous iron (Fe^2+^) to execute their catalytic activity (Tsukada et al., 2006; Perez-Perri et al., 2011). One of the family members, Jumonji C domain containing protein 6 (JMJD6), has emerged as a specialized lysyl hydroxylase (Webby et al., 2009). This was an important discovery since the only known lysyl hydroxylases at the time were PLOD (procollagen lysine, 2-oxoglutarate 5-dioxygenase) enzymes that catalyzed the lysine hydroxylation of another major ECM protein, collagen (Qi and Xu, 2018). We have reported that JMJD6 exhibits dual enzyme activity in the human placenta by mediating the lysyl hydroxylation and histone demethylation of von Hippel Lindau (VHL) tumor suppressor protein and gene, respectively (Alahari et al., 2018). JMJD6-mediated stabilization of VHL *via* oxygen-dependent lysyl hydroxylation promotes the degradation of hypoxia-inducible factor 1 alpha (HIF1A), a critical factor in the cellular oxygen sensing pathway (Alahari et al., 2015). Notably, while JMJD6 levels are elevated in preeclamptic placentae (Alahari et al., 2015), its *enzyme activity* is reduced due to placental hypoxia and diminished Fe^2+^ availability (Alahari et al., 2018).

Functionally, JMJD6 has been found to control migration, invasion, and proliferation of mesenchymal stem cells derived from adipose tissue (Shen et al., 2018), as well as in several breast cancer cell lines, and these biological actions were dependent on the activity of its catalytic JmjC domain (Lee et al., 2012; Poulard et al., 2015). Considering these studies, we hypothesized that impaired JMJD6 activity in PE contributes to the pathogenesis of this disease by affecting EVT differentiation along the invasive pathway *via* regulating FN lysine hydroxylation. While studies have reported high FN levels in PE, the mechanisms controlling FN trafficking and deposition in PE are unknown, prompting us to investigate FN processing and function in the human placenta under physiological and pathological conditions.

## Materials and Methods

### Placental Tissue Collection

All procedures were conducted in line with The Code of Ethics of the World Medical Association (Declaration of Helsinki) and Ethics Guidelines outlined by the Mount Sinai Hospital Research Ethics Board. Human placental tissue was collected by the Research Centre for Women’s and Infants’ Health (RCWIH) Biobank. Placentae were obtained from pregnancies complicated by early onset preeclampsia (E-PE, *n* = 54) and late-onset preeclampsia (L-PE, *n* = 13) diagnosed according to The American College of Obstetricians and Gynecologists (ACOG) guidelines American College of Obstetricians and Gynecologists (2013). Control placentae were obtained from normotensive age-matched pre-term control (PTC, *n* = 37) and term control (TC, *n* = 39) deliveries that did not show signs of disease. Table 1 summarizes the clinical parameters of the patient population. In addition, placentae were collected from the first trimester of pregnancy (5–12 weeks of gestation, *n* = 9) upon elective termination.

**TABLE 1:** Clinical parameters of patients.

	PTC	E-PE	TC	L-PE
	*n* = 37	*n* = 54	*n* = 39	*n* = 13
Gestational age at delivery	30.26 ± 2.96	29.114 ± 3.33	39.18 ± 1.00	39.50 ± 0.84
Birth weight (g)	1495.17 ± 596.87	1147.22 ± 692.16	3440.25 ± 365.90	3400 ± 721.11
Blood pressure	116.17 ± 4.75/	161.14 ± 13.14/	114.67 ± 5.10/	149.75 ± 10.50/
(mm Hg, S/D)	68.67 ± 5.71	97.14 ± 10.40	73.33 ± 5.90	90.25 ± 4.50
Proteinuria (g/day)	Absent	4 +	Absent	4+
Mode of	52% CS, 48%	88% CS, 12%	30% CS, 70%	50% CS,
delivery (%)	VD	VD	VD	50% VD

*Data are presented as mean ± standard error of the mean. PTC, Preterm Controls; PE, Preeclampsia; TC, Term Controls; S/D, Systolic/Diastolic; CS, Caesarian Section; VD, Vaginal delivery.*

### pMSC Isolation

Healthy first trimester (*n* = 9), TC and PTC (*n* = 8), and PE (*n* = 5) placentae were obtained immediately following delivery. Cleared villous tissue was washed twice in Hanks’ Balanced Salt Solution (HBSS). Tissue was initially digested twice in Dulbecco’s Modified Eagle Medium (DMEM) containing 0.05 mM trypsin (GIBCO 27250-018; Invitrogen, Carlsbad, CA, United States) and 0.008 mM DNase I (Sigma-Aldrich Corporation, St. Louis, MO, United States), for 30 min at 37°C. The remaining tissue was then incubated twice in a collagenase mixture (2 mg/ml collagenase, 0.1 mg/ml soybean trypsin inhibitor, 0.15 mg/ml DNase, and 1 mg/ml BSA dissolved in HBSS) at 37°C for 20 min. Between collagenase digestions, filtered supernatants underwent centrifugation at 1,500 rpm at room temperature (RT) for 5 min and cell pellets were resuspended in PBS containing 2% (v/v) fetal bovine serum (FBS). The cell suspension was layered onto a 5–70% Percoll (Sigma, St Louis, MO, United States) gradient and centrifuged at 1,400 *g* for 10 min at 4°C. The layer between 30 and 50%, containing pMSCs, was collected, centrifuged at 1,100 *g* for 15 min, and resuspended in DMEM. Cells were seeded for culture in DMEM plus 10% (v/v) FBS and 1% (v/v) penicillin/streptomycin. pMSCs were maintained at their respective normoxic conditions of either 8% O_2_ (5% CO_2_, 87% N_2__;_ TC pMSCs) or 3% O_2_ (5% CO_2_, 92% N_2_; 1st trimester and PE pMSCs). pMSCs were immunophenotyped *via* fluorescence-activated cell sorting (FACS) analyses to characterize their cell surface marker signatures.

### Lysosome Isolation

Lysosomes were isolated from PE (*n* = 8) and PTC (*n* = 8) placentae as described (Ermini et al., 2017). In brief, placental tissue chunks (approximately 0.5 g) were rinsed in saline and homogenized in 3 volumes of ice-cold buffer A (250 mM sucrose, 0.7 × 10^–3^ mM pepstatin, 1.1 × 10^–3^ mM leupeptin, 0.8 × 10^–3^ mM antipain, 80 × 10^–6^ mM aprotinin, and 10 mM Tris-HEPES, pH 7.4). Tissue homogenates were centrifuged at 5,860 *g* for 15 min and the harvested supernatant was centrifuged at 10,000 *g* for an additional 15 min followed by an additional spin at 25,000 *g* for 15 min to obtain lysosomes from the pellet.

### JMJD6 RNAi and Overexpression Studies

For RNAi knockdown studies, pMSCs were cultured to 50–60% confluency in 100-mm petri dishes and transfected with 30 nM of *JMJD6* Silencer^®^ siRNA duplexes (ID:23290, Cat # 4392420, Thermo Fisher Scientific, Waltham, MA, United States) or Silencer^®^ Negative Control siRNA (Cat #4390844, Thermo Fisher Scientific) using jetPRIME^®^ buffer (Polyplus transfection,^TM^ Illkirch, France) for 24 h. For overexpression studies, the p6352 MSCV-CMV-CMV-Flag-HA-JMJD6 plasmid [Addgene, Cambridge, MA, United States; Plasmid 31358 (Rahman et al., 2011)] was obtained as previously described (Alahari et al., 2015). Mutant JMJD6 plasmids were generated by site-directed mutagenesis against JMJD6 WT plasmids by mutating histidine 187 and aspartic acid 189 to alanine (H187A and D189A, respectively) (Alahari et al., 2018). Empty Vector control (EV) consisted of a MSCV PIG (Puro IRES GFP) (Plasmid 18751, Addgene) plasmid on an empty vector backbone. Plasmid (0.5 μg or 1.0 μg) was used for transfection studies in pMSCs. All plasmids were validated by sequencing.

### Deoxycholate-Solubility Assay

Placental tissue from PTC (*n* = 3), E-PE (*n* = 3), or TC and PE pMSCs were processed for deoxycholate fractionation. pMSCs were cultured to 60–70% confluency, and following appropriate treatments, soluble and insoluble FN protein extraction was adapted from a protocol reported by McKeown-Longo and Mosher (McKeown-Longo and Mosher, 1983).

### Cell Treatments

pMSCs were grown to ∼70% confluency in DMEM containing 10% (v/v) FBS in six-well plates. For inhibition of lysyl hydroxylase activity, cells were treated with 10 μM minoxidil, a general lysyl hydroxylase activity inhibitor (Murad and Pinnell, 1987) (Sigma-Aldrich, St. Louis, MO, United States) dissolved in 20 μl 95% (v/v) ethanol, and incubated for 48 h. Ethanol 95% (v/v) constituted the vehicle control. For inhibition of lysosomal activity or autophagosome fusion, respectively, pMSCs were exposed to 10 mM of NH_4_Cl (Sigma-Aldrich) dissolved in dH_2_O or alternatively 100 nM Baf A1 (Sigma) dissolved in DMSO for 24 h each. For iron supplementation, 25 or 100 μM of FAC (Sigma-Aldrich, St. Louis, MO, United States) or an equal volume of dH_2_O vehicle was added to cells for 24 h. For iron chelation, 50 μM of desferoxamine mesylate (DFO; Cayman Chemicals, Ann Arbor, MI, United States) or an equal volume of dH_2_O vehicle was added for 24 h. For inhibition of FN/α5β1 integrin binding, cells were treated with 1 μM of RGD peptide (Sigma-Aldrich, St. Louis, MO, United States) for 90 min. Cells were collected and harvested for immunofluorescence (IF) or Western Blotting (WB) analyses. For inhibition of protein synthesis, TC and PE pMSCs were cultured to 60–70% confluency in six-well plates and treated with 10 μg/ml of CHX (Sigma-Aldrich, St. Louis, MO, United States) or an equivalent volume of DMSO vehicle. Cells were collected and harvested for protein at various time points: 0, 3, 8, and 18 h following CHX exposure.

### Antibodies

Primary antibodies employed in IF and WB include mouse monoclonal anti-β-actin (ACTB) (sc-477778, 1:1,000, Santa Cruz), mouse monoclonal anti-integrin α5β1 (MAB1999, 1:200, Millipore Sigma), mouse monoclonal anti-α-tubulin (sc-8035, 1:1,000, Santa Cruz), mouse monoclonal anti-Calreticulin (CALR) (ab22683, 1:300, Santa Cruz Biotechnology), mouse monoclonal anti-CK7 (sc-70936, 1:200, Santa Cruz), rabbit polyclonal anti-collagen IV (COL IV) (ab19808, 1:200, Abcam), mouse monoclonal anti-Fibronectin (sc-8422, 1:1,000, Santa Cruz Biotechnology), rabbit polyclonal anti-Fibronectin (ab2413, 1:2,000, Abcam), mouse monoclonal anti-HLAG (ab52454, 1:200, Abcam), mouse monoclonal anti-JMJD6 (sc-28348, 1:500, Santa Cruz), rabbit polyclonal anti-JMJD6 (ab64575, 1:1,000, Abcam), rabbit monoclonal anti-Syntaxin 6 (CB4B2 #2869. 1:50, Cell Signaling Technology), and rabbit polyclonal anti-Vimentin (sc-5565, 1:200, Santa Cruz). HRP-conjugated secondary antibodies were obtained from Santa Cruz Biotechnology and used at a concentration of 1:2,000. WBs were scanned using a CanoScanLiDE20 image scanner (Canon Canada Inc. Mississauga, ON) and analyzed for densitometry.

### Western Blotting and Immunofluorescence

WB was conducted using 20–50 μg of placental tissue or, alternatively, pMSCs. IF staining was performed on pMSCs seeded onto coverslips. Both procedures were performed as previously described (Alahari et al., 2018; Farrell et al., 2019).

### *In vitro* Hydroxylation and MALDI-TOF Mass Spectrometry

Purified recombinant JMJD6 enzyme (100 or 200 ng) was incubated with 10 μg of synthetic FN peptides in reaction buffer [50 mM Tris-HCl (pH 7.9), 10 mM MgCl_2_, 50 mM KCl, 2 mM ascorbic acid, 1 mM α-ketoglutarate, and 50 μM Fe(NH_4_)_2_(SO_4_)_2_⋅6H_2_O in dH_2_O] for 1 h at 37°C in ambient air. The reaction mixtures were incubated and subjected to matrix-assisted laser desorption-ionization time-of-flight mass spectrometry (MALDI-TOF MS) at the Analytical Facility for Bioactive Molecules of the Hospital for Sick Children, Toronto, ON. Briefly, FN peptide-JMJD6 enzyme mixture was mixed with α-Cyano-4-hydroxycinnamic acid matrix (Cat no. 1405505, Sigma-Aldrich) in a 4:6 ratio. One microliter of the mixture was spotted onto the MALDI target plate and incubated at room temperature for 15 min until it crystallized. The plate was positioned into a time-of-flight tandem mass spectrometer (SCIEX TOF/TOF^TM^ 5800 System; SCIEX, Concord, Ontario) and subjected to ionization. Spectra were obtained using a Nd:YAG laser (337 nm) at 3 ns pulse width and 200 Hz firing rate. Data were acquired in the 0.5–2 kilodalton range using a positive-ion reflector mode. Each spectrum was generated from a pool of data obtained from multiple single laser shots at different positions in the sample spot. Spectra files were analyzed using Data Explorer software (SCIEX, Concord, ON, Canada).

### Quantification of Intracellular Total Iron and Labile Iron Pools

Intracellular iron content in TC and PE pMSCs was measured using a colorimetric Iron Assay Kit (Catalog # ab83366, Abcam Inc.). Briefly, 1.0 × 10^6^ cells were counted and homogenized using a Dounce Homogenizer in an acid buffer. To detect Fe^2+^ levels, an iron probe containing Ferene S, an iron chromogen, was added to the reaction. Sample absorbance was measured using a microplate reader at optical density 593 nm. For measurement of total iron levels, a reducing agent was added to the sample-probe mixture prior to detection. Fe^3+^ levels were calculated by subtracting Fe^2+^ content from total iron content. Concentrations were plotted against the standard curve, and iron content was normalized per 1.0 × 10^6^ cells.

For detection of the labile iron pool, cells were incubated with 2 μM Calcein AM dye (Thermo Fisher Scientific) for 30 min at 37°C in ambient air. Fluorescence was measured using a microplate reader at an excitation/emission spectrum of 488 nm/517 nm. Following initial readings, samples were immediately incubated with 100 μM DFO to quench iron-bound Calcein fluorescence, for 30 min at 37°C in ambient air. Fluorescence was once again measured at an excitation/emission spectrum of 488 nm/517 nm, and the concentration of the labile iron pool was determined as a function of the change in fluorescence following DFO chelation.

### ECM Isolation

Extracellular matrices were isolated as per a protocol outlined by Harris et al. (2018). In brief, pMSCs were seeded onto polystyrene culture dishes, eight-well Ibidi μ-slides, or, alternatively, on six-well plates with and without coverslips and allowed to grow for 4–5 days after reaching confluence. Cells were also transfected with JMJD6 plasmid vector or alternatively treated with 1 μM RGD peptide. Decellularization was initiated by rinsing cells twice in PBS, and once in wash buffer 1 [100 mM Na_2_HPO_4_, 2 mM MgCl_2_, and 2 mM EGTA (pH 9.6)]. This was followed by incubation in a hypotonic lysis buffer [8 mM Na_2_HPO_4_ and 1% NP-40 (pH 9.6)] for 15 min at 37°C. Fresh lysis buffer was replaced, and cells were further incubated for 20–40 min at 37°C. Cell lysis was monitored periodically by phase contrast microscopy. Following incubation, the lysis buffer was removed, and deposited matrix was rinsed several times in deionized water and stored in PBS at 4°C until use.

### Exosome Isolation and Uptake

Exosomes were isolated from conditioned media of TC and PE pMSCs cultured at their respective normoxic conditions of either 8% O_2_ (5% CO_2_, 87% N_2__;_ TC pMSCs) or 3% O_2_ (5% CO_2_, 92% N_2_; 1st trimester and PE pMSCs) as previously reported (Ermini et al., 2017). Briefly, conditioned media was collected and centrifuged at 12,000 *g* for 45 min to pellet larger vesicles followed by ultracentrifugation of the supernatant for 16 h at 100,000 *g*. The exosome pellet was suspended in PBS and filtered through a 0.22 μM filter. Exosome purity was verified by particle size analysis (NanoSight particle analyzer; Malvern Instruments Ltd, Malvern, United Kingdom). To test the optimal exosome uptake by HTR-8/SVneo cells, exosomes were labeled using the PKH67 Fluorescent Cell linker Kit (Sigma, PKH67GL-1KT), whereby a green fluorescent dye is stably incorporated into the exosome lipid membrane. HTR-8/SVneo cells were incubated with the labeled nanovesicles for 30 min, 1, 3, and 6 h, and optimal uptake time was found to be between 3 and 6 h. HTR-8/SVneo cells were exposed for 3 h to 2.0 × 10^6^ TC and PE exosomes and analyzed for IF.

### HTR-8/SVneo Cell Migration Assay and Time-Lapse Live Cell Imaging

HTR-8/SVneo cells were cultured in Roswell Park Memorial Institute (RPMI) 1640 media (Wisent Bioproducts, St. Bruno, QC, Canada) supplemented with 10% (v/v) FBS and 1% (v/v) penicillin/streptomycin, and maintained in standard conditions (21% O_2_, 5% CO_2_, and 74% N_2_) at 37°C. For *in vitro* migration assays, cells were seeded at a concentration of 30,000 cells/well onto previously extracted TC and PE pMSC-derived ECM on eight-well Ibidi μ-slides and grown to confluence. A straight “wound” was initiated within the cell monolayer by applying a light scratch using a sterile 200-μl pipette tip. Wound closure was imaged using an air immersion objective on a spinning disc confocal microscope (Leica Microsystems, Wetzlar, Germany), with images being captured every 15 min for 18 h. Rate of migration was quantified using Volocity Imaging software and determining the slope of wound area over time.

### Statistics

Statistical analyses were performed using GraphPad Prism 7.0 software (San Diego, CA, United States). When two groups were compared, an unpaired Student’s *t*-test or Mann–Whitney *U*-test was used. When comparing three or more groups, a one-way ANOVA followed by a Bonferroni *post-test* was used. Data are expressed as mean ± SEM (standard error of the mean).

## Results

### FN Protein Buildup in PE Is Due to Defective Turnover and Impaired Lysosomal Degradation

**FIGURE 1:**
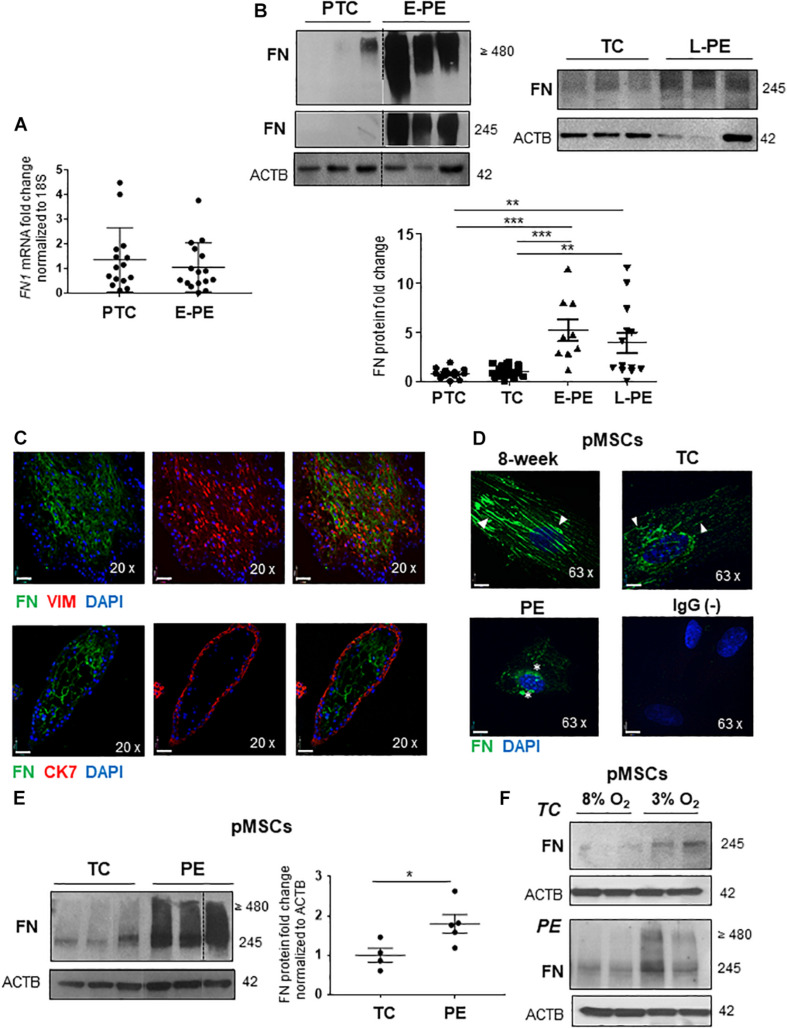
Fibronectin protein levels are increased in preeclamptic placentae and placental mesenchymal cells. **(A)** qPCR analysis of FN1 mRNA in age-matched PTC and E-PE placental tissue. *n* = 16 separate placentae per group. **(B)** Western blot of FN multimers (top panel) and monomers (bottom panel) in PTC vs. E-PE (top left panel; *n* = 13 and 11, respectively, per group) and TC vs. L-PE (top right panel; *n* = 22 and 13, respectively, per group) placentae. Associated densitometric analysis of FN across PTC, TC, E-PE, and L-PE placentae (bottom panel). **(C)** IF analysis of FN (green) and Vimentin (red) (top panel) and FN (green) with CK7 (red) (bottom panel) in chorionic villus sections from a first trimester placenta at 9 weeks of gestation. **(D)** IF for FN in pMSCs isolated from 8-week, TC (top panels) and PE (bottom left panel) placentae. Anti-rabbit IgG served as negative control (bottom right panel). Nuclei were counter-stained with DAPI. Arrowheads indicate FN fibrillar deposition in 8-week and TC pMSCs while stars point to FN clustering in PE pMSCs. Representative of three separate pMSC isolations per group. Scale bars at 20 × magnification represent 2.2 μM. **(E)** Western blot and corresponding densitometry for FN in TC and PE pMSCs. *n* ≥ 4 separate pMSC isolations per group. Data are presented as mean ± SEM. *P ≤ 0.05, **P ≤ 0.01, ***P ≤ 0.001 (unpaired Student’s *t*-test and one-way ANOVA with Bonferroni post-test). **(F)** Western blots for FN in TC and PE pMSCs exposed to 3% O_2_ and 8% O_2_.

We first investigated whether changes in FN levels in E-PE were a consequence of alterations in its gene and/or protein expression. qPCR analysis revealed that *FN1* mRNA levels were unchanged in E-PE vs. PTC placentae (Figure 1A). In contrast, WB for FN monomers (245; resolved under reducing conditions) and multimers (≥480; resolved under non-reducing conditions) showed markedly increased FN protein levels in E-PE relative to PTC placentae (Figure 1B). FN protein levels were also significantly increased in tissue lysates from L-PE placentae when compared to TC (Figure 1B). No changes in FN levels between PTC and TC were found (Figure 1B and Supplementary Figure 1A).

IF analysis of 1st-trimester placental villous tissue revealed a strong positive FN signal within the villous core, particularly within mesenchymal stromal cells (pMSCs) as indicated by vimentin (VIM)-positive staining; while no FN signal was detected in cytokeratin-7 (CK7)-positive trophoblasts (Figure 1C). A subset of FN-positive cells were observed in HLAG-expressing EVTs (Supplementary Figure 1B). Hence, in order to investigate FN processing in the human placenta under physiological and preeclamptic conditions, we isolated pMSCs from the stroma of 1st-trimester, TC and PE placentae as a surrogate tool to study placental FN homeostasis. Fluorescence-activated cell sorting (FACS) analyses confirmed the predominant expression of mesenchymal stromal cell surface markers CD29, CD73, CD90, and CD105 and lack of hematopoietic markers, CD34 and CD45, in representative 7-week and PE pMSCs (Supplementary Figure 1C). IF for FN in primary isolated pMSCs revealed striking differences in FN appearance in PE pMSCs when compared to 1st-trimester (8-week) and TC cells (Figure 1D). Particularly, while FN existed in a fibrillar/elongated cell-associated network and localized to the peri-cellular region in 1st-trimester and TC pMSCs, it was clustered and disorganized in pMSCs from PE pregnancies (Figure 1D). In addition to alterations in its deposition, FN monomer and multimer protein levels were significantly increased in PE pMSCs (Figure 1E), contributing to the overall overexpression of FN in PE placentae. Exposure of either TC or PE pMSCs to 3% O_2_ augmented FN levels (Figure 1F). Collectively, these data indicate impaired FN deposition and signaling in PE pMSCs that is dependent on the low-oxygenated environment typical of PE pathologies.

**FIGURE 2:**
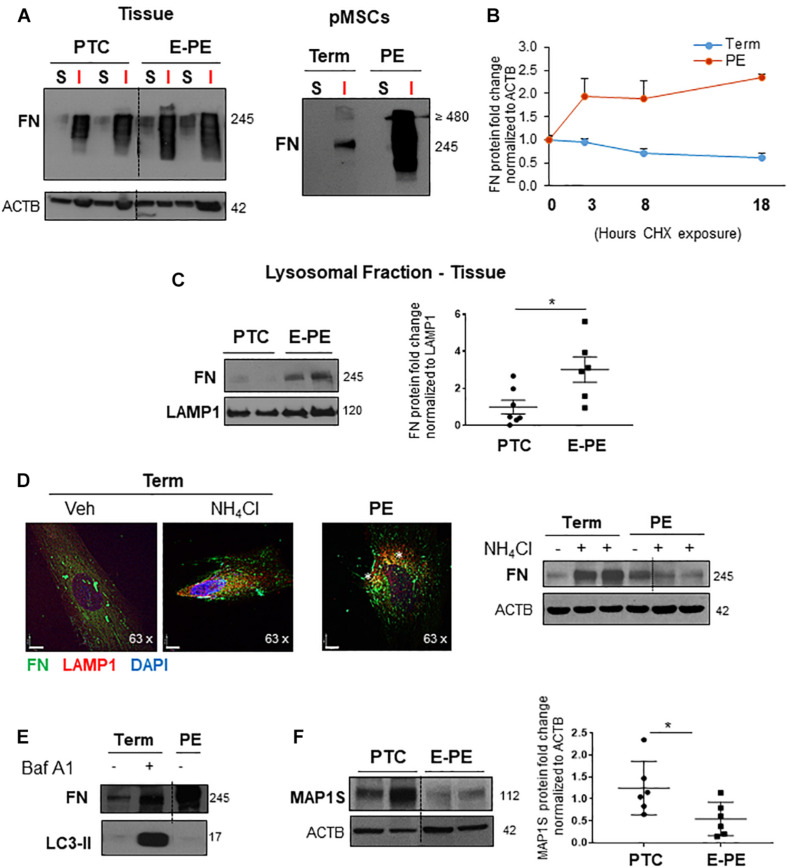
Fibronectin matrix turnover and degradation are impaired in preeclampsia. **(A)** Western blot for FN in the matrix-soluble and -insoluble protein fractions from PTC (*n* = 3) and E-PE (*n* = 3) placental tissue (left panel) or TC and PE pMSCs (right panel) following sodium deoxycholate extraction. Representative of two separate pMSC isolations. **(B)** FN matrix turnover in TC and PE pMSCs, normalized to Actin (ACTB) over a time course of 24 h upon exposure to cycloheximide (CHX). *n* = 3 separate pMSC isolations. **(C)** Western blots (left panel) and corresponding densitometry (right panel) for FN in the lysosomal fraction isolated from PTC and PE placentae. The lysosomal marker, LAMP1, served as loading control. *n* = 7 separate placentae per group. **(D)** (left panel) IF for FN (green) and LAMP1 (red) in TC pMSCs exposed to NH_4_Cl, compared to PE pMSCs. Nuclei were counter-stained with DAPI. Scale bars at 63 × magnification represent 7 μM. Representative of three separate pMSC isolations. **(D)** (right panel) Western blot for FN in TC and PE pMSCs exposed to 10 μM NH_4_Cl for 24 h; − = H_2_O Vehicle, + = 10 μM NH_4_Cl. *n* = 3 separate pMSC isolations. **(E)** Western blot for FN in TC pMSCs exposed to 100 nM Bafilomycin for 24 h and in PE pMSCs; V = DMSO Vehicle, B = 100 nM Bafilomycin. **(F)** Western blot and corresponding densitometry for MAP1S in PTC and PE placental tissue. *n* = 6 separate placentae per group. Data are presented as mean ± SEM. *P ≤ 0.05 (unpaired Student’s *t*-test).

The gold standard assay for demonstrating FN matrix assembly is the irreversible conversion from sodium deoxycholate (DOC) solubility to insolubility (McKeown-Longo and Mosher, 1983). Hence, we extracted protein from TC and PE pMSCs as well as from PTC and E-PE placental tissue using a DOC fractionation assay and separated soluble cell-associated, and insoluble ECM-associated protein fractions. Following WB for FN, we observed that (1) FN is predominantly present in the insoluble protein fraction and (2) insoluble FN is enriched in E-PE tissue and in PE pMSCs (Figure 2A), indicating impaired FN turnover. For further confirmation, we next assessed FN protein half-life using cycloheximide (CHX), an inhibitor of protein synthesis. Employing a CHX-chase assay (treatment durations of 3, 8, and 18 h), we found that in TC pMSCs, FN levels were indeed reduced over the course of 18 h, while in PE, they remained unchanged throughout the duration of CHX exposure, indicating impaired degradation (Figure 2B).

Next, we reasoned that high FN content in PE may in part be due to deficiency in its degradation. We first examined FN content in lysosomes isolated from preeclamptic placental tissue lysates and found significantly augmented lysosomal FN content in PE (Figure 2C). IF analysis of FN distribution upon exposure of TC pMSCs to the lysosomotropic agent ammonium chloride (NH_4_Cl; increases lysosomal pH) demonstrated intracellular accumulation of FN, as well as enhanced co-localization with the lysosomal marker, LAMP1 (Figure 2D, left panel). Importantly, NH_4_Cl treatment of TC pMSCs mimicked FN spatial distribution in PE pMSCs (Figure 2D, right panel), further underscoring the lack of lysosomal FN turnover in PE. IF findings were corroborated by WB for FN following NH_4_Cl treatment of TC and PE pMSCs showing FN accumulation in controls and no changes in PE (Figure 2D, right panel). Likewise, exposure to Bafilomycin A1 [Baf A1, a vacuolar type H(1)-ATPase inhibitor of lysosomal acidification (Mattsson et al., 1991)] resulted in FN buildup in TC pMSCs, like that found in untreated PE pMSCs (Figure 2E). Corresponding WB for the autophagy substrate, microtubule-associated protein 1A/1B-light chain 3 (LC3-II) showed a marked accumulation upon Baf A1 treatment, confirming autophagy flux inhibition (Figure 2E). Analysis of levels of MAP1S (Microtubule Associated Protein 1S), a known autophagy activator for FN, revealed a significant reduction in PE placentae, further underscoring a defect in the autophagy clearance pathway in PE (Figure 2F).

### The Oxygen Sensor, JMJD6, Controls FN Content in Placental Mesenchymal Cells

**FIGURE 3:**
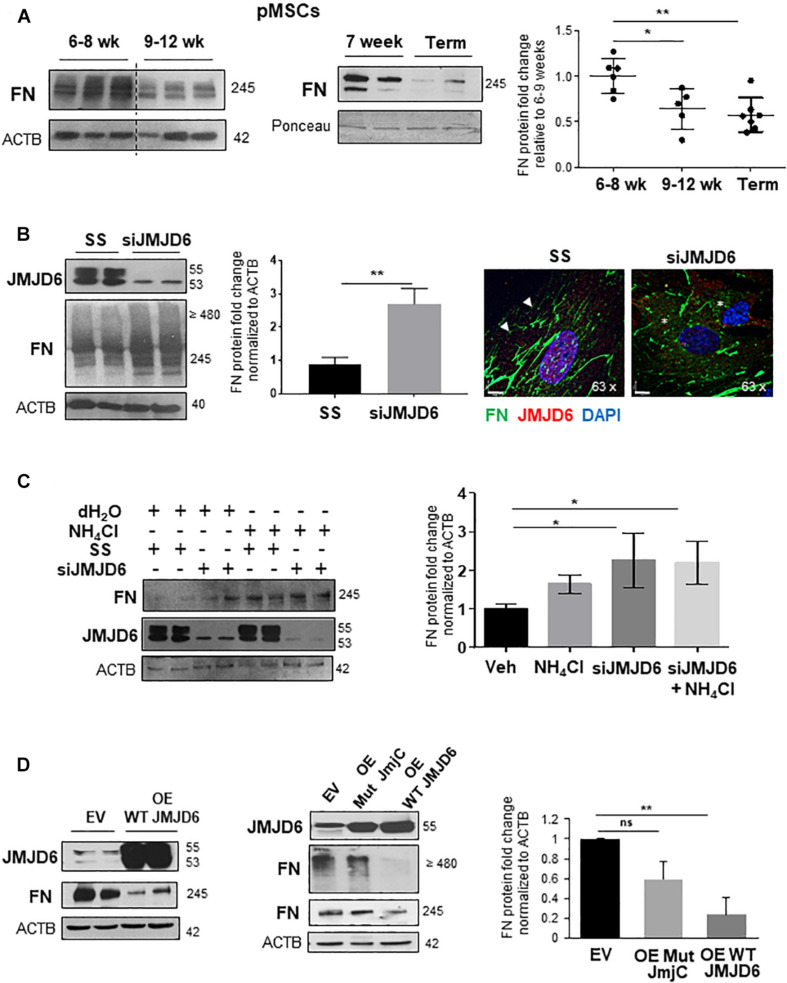
The oxygen sensor, JMJD6, is required for fibronectin matrix formation. **(A)** Western blot and corresponding densitometry for FN in the first trimester (6–8 weeks, 9–12 weeks, and TC pMSCs). *n* = 3 separate pMSC isolations. **(B)** (left panel) Western blot and densitometry for JMJD6 and FN in TC pMSCs upon *JMJD6* siRNA knockdown (siJMJD6). *n* = 3 separate pMSC isolations. **(B)** (right panel) IF for FN (green) and JMJD6 (red) following siJMJD6 in TC pMSCs. Nuclei were counter-stained with DAPI. Scale bars at 63 × magnification represent 7 μM. Representative of three separate pMSC isolations. **(C)** Western blot and corresponding densitometry for FN and JMJD6 upon *JMJD6* siRNA knockdown (siJMJD6) in concert with NH_4_Cl exposure of TC pMSCs. *n* = 3 separate pMSC isolations. **(D)** Western blots and associated densitometry for JMJD6 and FN in TC pMSCs overexpressing (OE) WT JMJD6 (left panel) or mutated JMJD6 constructs (Mut JmjC; right panel). EV, vehicle control. *n* = 3 separate pMSC isolations, Actin (ACTB) served as loading control. Data are presented as mean ± SEM. *P ≤ 0.05 **P ≤ 0.01 (unpaired Student’s *t*-test; one-way ANOVA and Bonferroni *post-test*).

Placental hypoxia is central to the pathogenesis of PE (Soleymanlou et al., 2005), and *in vitro* exposure to hypoxia has been reported to upregulate FN levels in cytotrophoblast cells (Genbacev et al., 2001). Hence, we examined whether FN expression of pMSCs varied with changes in oxygen tension *in vivo*. Exposure of pMSCs from early 1st trimester (6–8 weeks) and 9–12 weeks or TC placentae to their respective normoxic conditions, namely, 3 and 8% O_2_, revealed that FN levels were significantly higher at 6–8 weeks, representing a gestational window characterized by physiological hypoxia *in vivo* (Figure 3A; Ietta et al., 2006). To uncover a potential molecular mechanism for this phenomenon, we next investigated whether the oxygen sensor JMJD6, which we have previously reported to be upregulated but inactive in PE (Alahari et al., 2018), regulated FN expression in pMSCs. Following siRNA knockdown of *JMJD6* in TC pMSCs kept at 8% O_2_, FN levels were markedly elevated (Figure 3B, left panels). To determine whether loss of *JMJD6* influenced FN spatial distribution in control TC pMSCs, we performed IF for FN upon siRNA knockdown of *JMJD6* and found pericellular accumulation and disorganized FN fibril deposition (Figure 3B, right panels), reminiscent of the FN appearance in PE pMSCs.

To understand whether JMJD6 is dispensable for FN lysosomal degradation, we performed siRNA knockdown of *JMJD6* in TC pMSCs exposed to NH_4_Cl to impede lysosomal function. pMSCs exposed to NH_4_Cl following *JMJD6* loss did not demonstrate further lysosomal retention compared to silencing alone (Figure 3C). However, silencing of JMJD6 augmented the effect of NH_4_Cl on FN accumulation, suggesting that JMJD6 in part contributes to FN lysosomal degradation (Figure 3C).

We next questioned whether JMJD6 acts on FN *via* the Jumonji C domain that is the key site of catalytic activity (Chang et al., 2007). Hence, we transfected TC pMSCs with either wild-type (WT) or mutated JmjC constructs containing two point mutations in the JmjC domain (Alahari et al., 2018), thereby abolishing its enzyme function. WT JMJD6 overexpression significantly reduced FN protein levels (Figure 3D, left panel). In contrast, overexpression of Mut JmjC did not significantly affect the levels of both monomeric and dimeric/multimeric forms of FN, confirming the importance of the JmjC domain in mediating the effects of JMJD6 (Figure 3D, right panels).

### FN Is a Putative Target of JMJD6-Mediated Lysyl Hydroxylation in pMSCs

**FIGURE 4:**
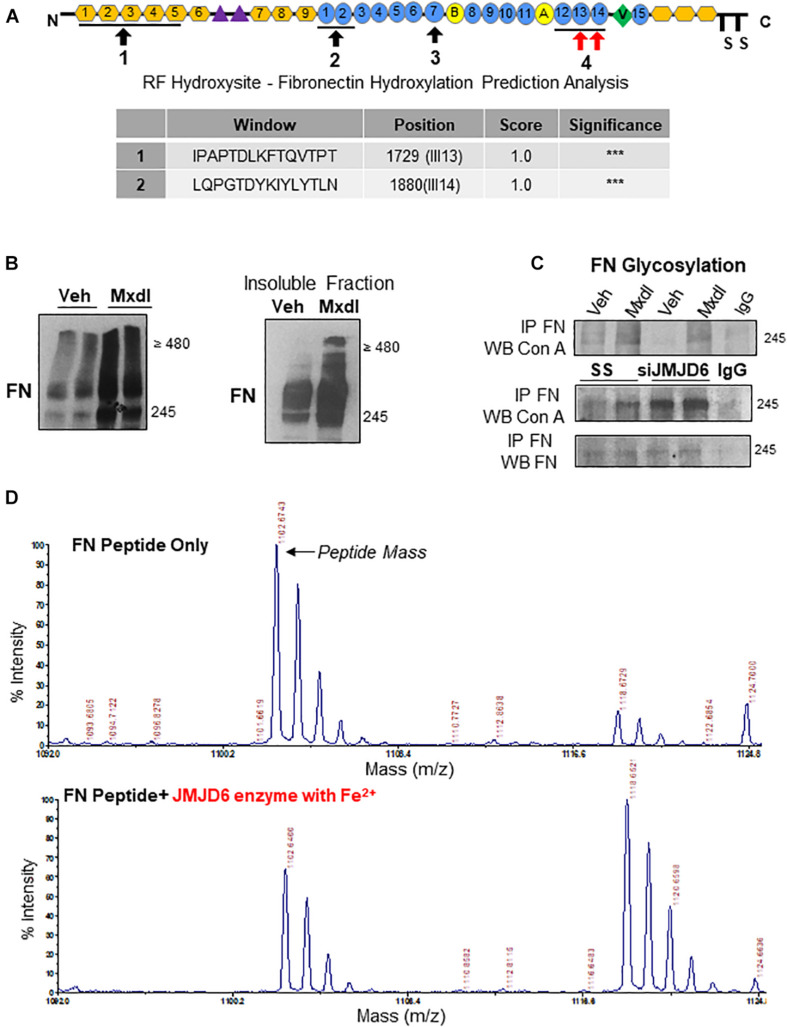
Fibronectin is a novel target of JMJD6-mediated lysyl hydroxylation and glycosylation. **(A)** Schematic of FN protein indicating sites involved in mediating FN–FN association during fibrillogenesis (black arrows). Table of FN protein sequences within the afore-indicated sites with a 100% probability of having lysine residues that are hydroxylated (red arrows). Sequences and probabilities were determined using the RF Hydroxysite prediction software. “***” represents the degree of highest probability of lysyl hydroxylation (100%). **(B)** (left panel) Western blot for FN following exposure of TC pMSCs to 10 μM minoxidil (Mxdl) for 48 h. *n* = 2 separate pMSC isolations. **(B)** (right panel) Western blot for FN in the insoluble matrix-associated protein fraction in TC pMSCs exposed to Mxdl. Representative of two separate pMSC isolations. **(C)** Western blot for FN and concanavalin A (Con A) following immunoprecipitation of FN upon Mxdl exposure (top panel) and *JMJD6* siRNA knockdown (siJMJD6, bottom panel) in TC pMSCs (*n* = 3 separate pMSC isolations). Anti-rabbit IgG served as a negative control. **(D)** Representative mass spectra for FN peptide sequence 2 indicating the original peptide mass (top panel) and a 16-mass unit shift upon incubation with recombinant JMJD6 enzyme (200 ng) in the presence of ferrous iron (Fe^2+^; bottom panel).

*In silico* analysis of the FN protein revealed the presence of four key domains important for FN–FN binding and dimerization (Figure 4A, top panel). Using a hydroxylation prediction software (RF Hydroxysite), we determined that among these sites, two domains contained lysine residues that had a significant likelihood of being targeted for hydroxylation (Figure 4A, bottom panel, indicated by red arrows in the top panel). Considering JMJD6 functioning as a lysyl hydroxylase (Unoki et al., 2013), we investigated whether FN is regulated by JMJD6 through such mechanism. Exposure of TC pMSCs to the general lysyl hydroxylase inhibitor, minoxidil, resulted in accumulation of FN monomers and multimers (Figure 4B, left panel), and this occurred predominantly in the insoluble, matrix-associated fraction (Figure 4B, right panel). Lysyl hydroxylation of collagen, another ECM protein, has been shown to be a prerequisite for its subsequent glycosylation and fibrillar assembly (Gjaltema and Bank, 2017). Hence, we next examined whether JMJD6 hydroxylation of FN affected FN glycosylation, an event that precedes its maturation and biological function (Jones et al., 1986). As a readout, we first studied FN association to the sugar-binding lectin Concanavalin A (Con A) by co-immunoprecipitation analysis following minoxidil treatment. We found that minoxidil exposure of TC pMSCs resulted in elevated FN-Con A binding (i.e., increased glycosylation) (Figure 4C, top panel). Like lysyl hydroxylase inhibition with minoxidil, FN glycosylation was also increased upon *JMJD6* siRNA knockdown in TC pMSCs (Figure 4C, bottom panel), suggesting that JMJD6-mediated lysyl hydroxylation of FN negatively impacts on its glycosylation.

To conclusively demonstrate that JMJD6 is capable of hydroxylating FN, we generated synthetic peptides (FN_172__6__–__1733_ and FN_187__7__–__1884_) spanning the two high probability sites on FN (Figure 4A, top panel) and performed an “*in vitro* hydroxylation” reaction with recombinant JMJD6 enzyme in the presence of the JMJD6 co-factor, Fe^2+^. Upon subjecting the reaction products to MALDI-TOF mass spectrometry, we detected a 16-dalton shift in mass of one of the FN peptides (FN_187__7–1884_) in the presence of JMJD6 enzyme, corresponding to the addition of a single hydroxyl group (Figure 4D). When the enzymatic reaction was performed in the absence of Fe^2+^, the shift in mass, as well as dissolution of the primary peptide mass peak, was much less pronounced (Supplementary Figure 2).

### Exposure of PE pMSCs to Hinokitiol Restored FN Matrix Formation

**FIGURE 5:**
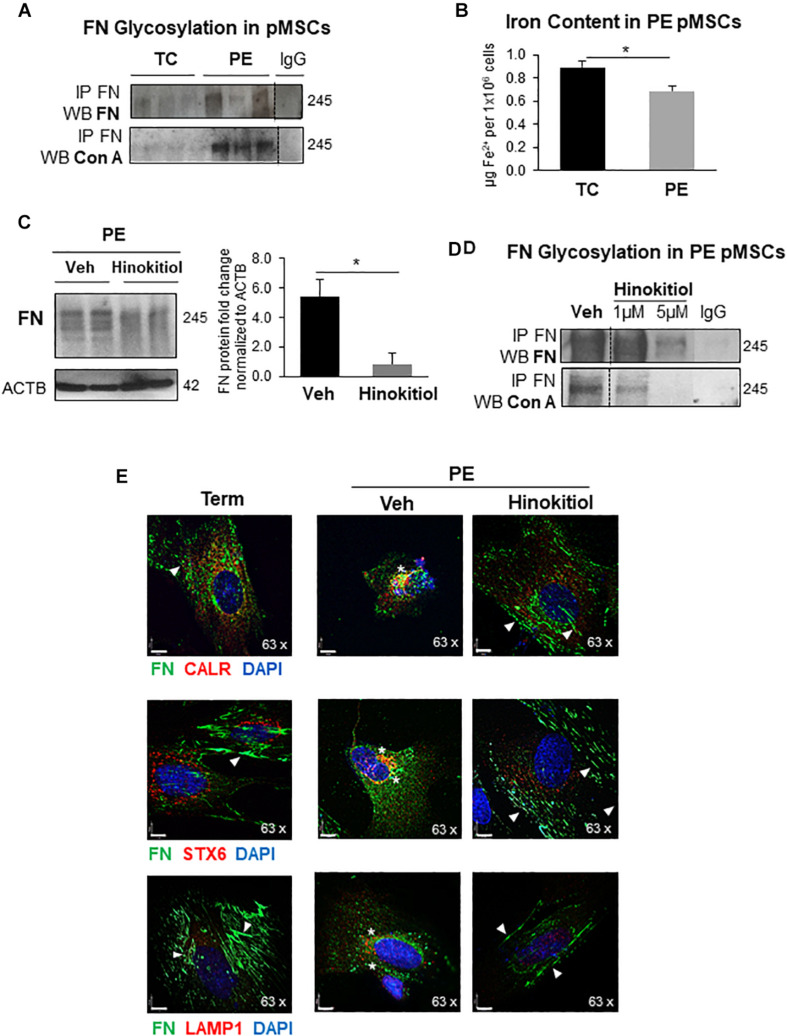
Exposure of preeclamptic pMSCs to Hinokitiol rescues FNglycosylation and matrix deposition. **(A)** Western blot for FN and Con A following immunoprecipitation of FN in TC and PE pMSCs. *n* = 3 separate pMSC isolations. **(B)** Intracellular Fe^2+^ content in TC and PE pMSCs. *n* = 4 separate pMSC isolations performed in duplicate. **(C)** Western blot and densitometry for FN in PE pMSCs upon exposure to 1 μM Hinokitiol. *n* = 4 separate pMSC isolations. **(D)** Western blot for FN and Con A following immunoprecipitation of FN in PE pMSCs exposed to 1 μM Hinokitiol. Representative of two separate pMSC isolations. **(E)** IF for FN (green) and the organelle markers CALR (ER), STX6 (Golgi), and LAMP1 (lysosomes) in red (top, middle, and bottom panels, respectively) following 1 μM Hinokitiol exposure. In IF images, arrowheads indicate FN fibrillar deposition while stars show FN clustering. Nuclei were counterstained with DAPI. Scale bars at 63× magnification represent 7 μM. Data are presented as mean ± SEM. *P ≤ 0.05 (Unpaired Student’s *t*-test).

In agreement with reported impairment of JMJD6 activity in PE, FN glycosylation, probed with Con A, was increased in PE pMSCs vs. TC pMSC controls (Figure 5A). JMJD6 activity is intimately linked not only to oxygen availability but also to cellular Fe^2+^ status. Corresponding to the reduced intracellular iron content in PE placentae (Alahari et al., 2018), we also found less Fe^2+^ in PE pMSCs relative to TC pMSCs controls (Figure 5B). Therefore, we next examined whether FN defects in PE pMSCs can be rescued with Hinokitiol, a natural compound that was recently shown to promote unidirectional iron transport in murine tissue lacking cellular iron transporters (Grillo et al., 2017). Following time and dose optimization, treatment of PE pMSCs with 1 μM Hinokitiol for 24 h resulted in a significant decrease in FN protein levels (Figure 5C). Importantly, Hinokitiol exposure of PE pMSCs not only decreased overall FN content but also attenuated FN glycosylation (Figure 5D). Similarly, treatment of TC pMSCs with Hinokitiol also resulted in FN reduction (Supplementary Figure 3A). IF analysis of FN and the ER marker, calreticulin (CALR) upon Hinokitiol treatment of PE pMSCs demonstrated a marked redistribution of FN aggregates in proximity to the ER toward to a more fibrillar appearance reminiscent of TC pMSCs (Figure 5E, top left panel). Similarly, Hinokitiol exposure reduced FN accumulation in the Golgi [co-localization with syntaxin 6 (STX6)] and lysosomes (Figure 5E, middle and bottom right panels) in PE, resulting in an elongated, fibrillar pattern of deposition similar to control pMSCs.

**FIGURE 6:**
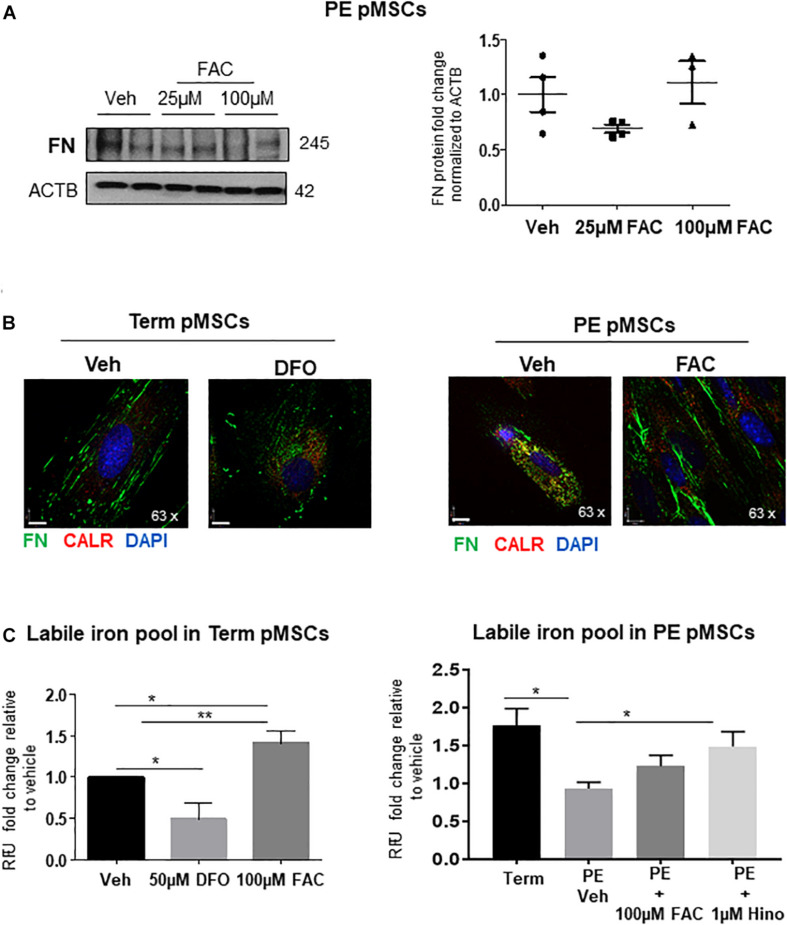
Iron supplementation of PE pMSCs improves FN deposition. **(A)** Western blot and corresponding densitometry for FN in TC pMSCs (left panels) and PE pMSCs (right panels) treated with 25 or 100 μM FAC. *n* ≥ 3 separate pMSC isolations per group performed in duplicate. **(B)** IF for FN (green) and CALR (red) in TC pMSCs exposed to 50 μM of the iron chelator, DFO (left panel), and in PE pMSCs exposed to 100 μM FAC (right panel). Scale bars at 63× magnification represent 7 μM. Representative of three separate pMSC isolations per group. **(C)** Quantification of the labile iron pool in TC pMSCs exposed to 50 μM DFO or 100 μM FAC (left panel) and in PE pMSCs exposed to 100 μM FAC and 1 μM Hinokitiol (right panel). Values are expressed as fold change of relative fluorescence units (RFU; measured at 488 nM excitation/517 nM emission) over vehicle controls. *n* = 4 and 3 separate pMSC isolations, respectively, per group. Data are presented as mean ± SEM. *P ≤ 0.05, **P ≤ 0.01 (one-way ANOVA and Bonferroni *post-test*).

In light of the observation that Hinokitiol exposure improved FN deposition, we employed an alternate method of iron supplementation to PE pMSCs by treating them with ferric ammonium citrate (FAC). Addition of 25 or 100 μM FAC to PE pMSCs had no discernible impact on FN protein levels (Figure 6A). Accompanying IF analysis showed that addition of 100 μM FAC restored the fibrillar, pericellular deposition of FN (Figure 6B, right panels). Interestingly, addition of the iron chelator DFO (Steinmetz et al., 1980) to TC pMSCs resulted in a FN appearance like that seen in PE pMSCs (Figure 6B, left panels), further highlighting the significance of iron in controlling FN deposition in placental mesenchymal cells. To further assess whether addition of FAC or Hinokitiol influenced intracellular iron content, we performed a Calcein-AM fluorometric assay to quantify the labile (useable) iron pool in TC and PE pMSCs. As anticipated, addition of FAC significantly increased the labile iron pool in TC control pMSCs (Figure 6C, left panel). In contrast, DFO significantly reduced the useable iron pool in TC pMSCs. Preeclamptic pMSCs had significantly less labile iron than TC pMSC controls (Figure 6C, right panel), in line with our observation of diminished Fe^2 +^ content in PE pMSCs (Figure 5B). Exposure of PE pMSCs to Hinokitiol markedly increased the labile iron pool in the cells, while FAC treatment had a less pronounced effect (Figure 6C, right panel). Together, these findings suggest that Hinokitiol restores FN deposition in PE pMSCs by increasing the useable iron pool and, thereby, JMJD6 hydroxylase activity.

### Extravillous Trophoblast Cell Migration Is Impaired on Preeclamptic pMSC-Derived ECM

**FIGURE 7:**
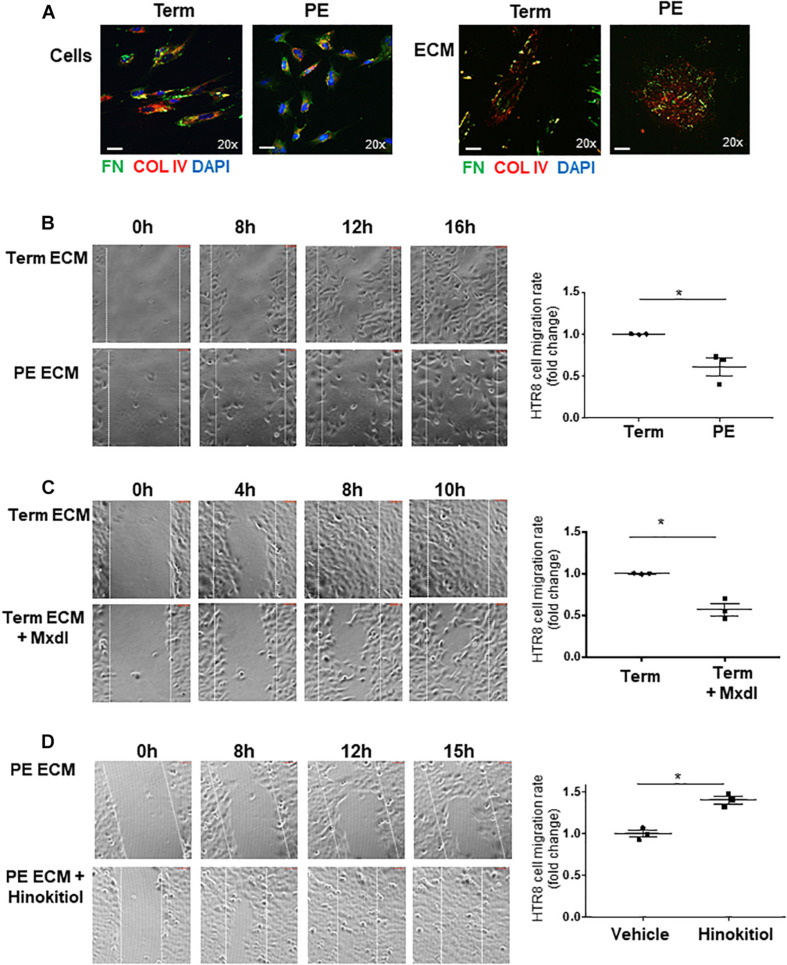
Defects in ECM deposited by PE pMSCs impedes extravillous trophoblast cell migration *in vitro*. **(A)** IF analysis of FN (green) and COL IV (red) in TC and PE pMSCs prior to (top panel) and following ECM extraction (bottom panels). Scale bars at 20× magnification represent 2.2 μM. Representative of three separate pMSC isolations. **(B)** Images and associated quantification (expressed as a migration index fold changes relative to control) of HTR-8/SVneo cell migration over time on TC or PE pMSC-deposited ECM, captured by time-lapse microscopy; *n* = 3 separate pMSC isolations per group. **(C)** Snapshot images and quantification of HTR-8/SVneo cell migration over time on ECM deposited by TC pMSCs treated with vehicle control (95% EtOH) or minoxidil (Mxdl). *n* = 3 separate pMSC isolations. **(D)** Representative images and quantification of HTR-8/SVneo cell migration over time on ECM deposited by PE pMSCs treated with control vehicle (DMSO) or 1 μM Hinokitiol, captured by time-lapse microscopy; *n* = 3 separate pMSC isolations. Data are presented as mean ± SEM. *P ≤ 0.05 (unpaired Student’s *t*-test).

pMSCs have been reported to trigger EVT migration/invasion (Chen et al., 2013; Chen, 2014). Considering the defects in FN assembly in PE pMSCs, we next investigated the functional significance of pMSC-deposited FN and its ability to support EVT cell migration *in vitro*. Hence, we isolated ECM from TC and PE pMSCs and characterized them by WB for the presence of FN and collagen type IV (COL-IV) (Supplementary Figure 3A), and by phase contrast microscopy to visualize overall ECM structure (Supplementary Figure 3B). WB for the cytoskeletal marker, α-Tubulin, confirmed the absence of cellular components in the deposited ECM after decellularization (Supplementary Figure 3A). Similarly, IF analyses for FN and COL-IV prior to and following decellularization revealed distinct patterns of deposition in the cellular- and matrix-associated fractions between TC and PE pMSCs (Figure 7A). The absence of cells was also confirmed by the lack of nuclear DAPI signal (Figure 7A, bottom panels). To study EVT migration, we employed HTR-8/SVneo cells, a well-established placental EVT cell line commonly used to investigate migratory events (Lee et al., 2001). After seeding HTR-8/SVneo cells onto the acellular matrix, a scratch wound was initiated in the confluent monolayer and time-lapse imaging of wound closure was performed as an index of cell migration. Our data revealed that while ECM derived from both groups of pMSCs was able to sustain HTR-8/SVneo cell proliferation *in vitro*, HTR-8/SVneo cells cultured on the matrix deposited by PE pMSCs migrated significantly slower when compared to cells cultured on the acellular TC matrix (Figure 7B and Supplementary Movies 1A,B). These data suggest that the ECM deposited by PE pMSCs is deficient in supporting EVT migration. Next, the role of lysyl hydroxylation on ECM deposition was assessed by treating TC pMSCs with minoxidil prior to decellularization. Our data show that HTR-8/SVneo cells migrated markedly slower on matrix deposited by TC pMSCs treated with minoxidil compared to matrix deposited by vehicle-treated TC pMSCs (Figure 7C and Supplementary Movies S2A,B), suggesting that lysyl hydroxylation contributes to proper ECM, including FN, deposition that supports EVT migration. Furthermore, HTR-8/SVneo cells on ECM derived from TC pMSCs that were transfected with JMJD6 vector migrated at a slower rate compared to empty vector controls (Supplementary Figure 4). To further confirm the involvement of pMSC-derived FN in EVT migration, HTR-8/SVneo cells were cultured on pMSC-derived matrices that were treated with a RGD peptide (blocks FN binding to its α5β1 integrin receptor) followed by IF for FN and α5β1 integrin. Pre-treatment of pMSC matrix with RGD peptide markedly reduced FN ligand/receptor levels and their association (Supplementary Figure 5A). Also, HTR-8/SVneo migratory capacity was markedly reduced on RGD matrices (Supplementary Figure 5B).

Studies have reported that pMSC-derived ECM can modulate EVT migration and invasion *in vitro* through paracrine interactions (Chen and Aplin, 2003; Chen, 2014). Furthermore, emerging evidence indicates that pMSCs communicate *via* exosomes with neighboring EVT cells, thereby triggering their migration and invasion (Chen et al., 2020). Hence, we reasoned that pMSC exosomal cargo communicates with EVT, affecting their cell behavior. Exosomes isolated from TC and PE pMSC-conditioned media were validated by Nanosight tracker analysis (Supplementary Figure 6A). WB analysis showed increased FN cargo in PE pMSCs exosomes relative to those from TC pMSCs (Supplementary Figure 6B). We first tested the uptake of pMSC-derived exosomes by HTR-8/SVneo using a PKH67 Fluorescent Cell Linker Kit that incorporates a green fluorescent dye into the isolated exosomes. IF analysis demonstrated that HTR-8/SVneo cells readily uptake exosomes as early as 30 min of exposure (Supplementary Figure 6C). IF analysis showed that exposure of HTR-8/SVneo cells to TC and PE exosomes resulted in augmented FN positive signal, particularly after PE exosome treatment (Supplementary Figure 6D).

Finally, we examined whether impaired HTR-8/SVneo migration on matrix deposited by PE pMSCs can be corrected with Hinokitiol. We observed that following wound initiation, HTR-8/SVneo cells migrated significantly faster on matrix deposited by PE pMSCs treated with Hinokitiol compared to that from PE pMSCs treated with vehicle (Figure 7D and Supplementary Movies 3A,B).

## Discussion

FN is a multi-faceted molecule mediating integral aspects of cell behavior. Despite its critical involvement in orchestrating essential cell differentiation events in the placenta, there is a major gap in knowledge on the mechanisms controlling FN homeostasis in normal and pathological conditions. In the present study, we demonstrate placental accumulation of unprocessed FN protein due to impediments in its lysosomal degradation. Furthermore, we provide new evidence that FN protein processing is markedly impaired in mesenchymal cells from preeclamptic placentae, owing to defects in regulation by the O_2_- and Fe^2+^-dependent enzyme, JMJD6. Exposure to Hinokitiol restored FN homeostasis in these cells by correcting JMJD6 function and, as a result, EVT migration.

### The Oxygen Sensor, JMJD6, Is a Critical Regulator of FN Processing That Is Impaired in PE

JMJD6 loss in TC pMSCs faithfully mimicked FN disorganized and clustered appearance in PE pMSCs, implying that impaired JMJD6 activity in PE contributes to dysregulated FN homeostasis. Our mass spectrometric data uncover FN as a new target of JMJD6-mediated lysyl hydroxylation. Furthermore, we showed that this post-translational modification of FN is necessary for subsequent glycosylation. Studies with the lectin Con A revealed that overall N-glycosylation of cellular FN was elevated upon *JMJD6* loss in TC pMSCs, similar to that found in PE pMSCs (Alahari et al., 2018). Since N-glycosylation primarily occurs in the ER, our data suggest that JMJD6 controls FN processing post-translationally, in the early stages of its biosynthesis. Given the significance of lysyl hydroxylation for subsequent post-translational modifications in collagen (Gjaltema and Bank, 2017), it is likely that deficient lysyl hydroxylation of FN by JMJD6 (in the ER) leads to elevated levels of glycosylated FN, which, in turn, is secreted into extracellular space. Alternatively, this glycosylated FN is targeted to the lysosomes where it accumulates and fails to be degraded appropriately. In addition to its lysyl hydroxylase role, JMJD6 executes histone arginine demethylation and was recently reported to have intrinsic tyrosine kinase activity on histone H2A.X target (Liu et al., 2019). However, given that we found no changes in *FN1* mRNA in preeclamptic placentae, it is unlikely that histone arginine demethylation plays a major role in controlling FN protein levels in the human placenta. Nevertheless, future studies may unravel a direct regulation of *FN1* gene by JMJD6-mediated histone demethylation or tyrosine-kinase dependent phosphorylation in other systems.

In the current study, we found that the effects of JMJD6 on FN are predominantly mediated through the catalytic JmjC domain that is central to its lysyl hydroxylase function (Chang et al., 2007). Of interest, we noted that overexpression of both WT JMJD6 and JMJD6 with mutations in the JmjC domain resulted in a profoundly disrupted matrix, but in contrast to WT overexpression, mutated JMJD6 did not significantly affect the amount of total FN protein. This suggests that the JmjC domain is necessary not only for maintenance of overall FN protein homeostasis, but also for facilitating the proper conversion of soluble FN into its insoluble, matrix-associated form. Furthermore, JMJD6 overexpression in pMSC-derived ECM abrogated HTR-8/SVneo cell migration on the matrix, suggesting that regulation of the JMJD6-FN rheostat in pMSCs is critical for EVT cell motility. Recently, Miotti et al. (2017) reported that besides its typical localization in the nucleus, JMJD6 was also secreted into the ECM deposited by human tumor cell lines where it was found to interact with collagen type I (COL-I) and influence subsequent collagen–FN interaction. Interestingly, the authors proposed that the function of JMJD6 in the ECM is independent of its JmjC domain (Miotti et al., 2017), suggesting that JMJD6 may also *indirectly* affect FN function. One report suggests that JMJD6 interacts with the epigenetic reader, bromodomain-containing protein 4 (BRD4) that is a potent transcriptional repressor of lysosomal function and the autophagy machinery (Liu et al., 2013; Sakamaki et al., 2017). Future studies will investigate the potential epigenetic involvement of JMJD6 in the lysosomal–autophagy system and its direct contribution to FN degradation.

### FN Trafficking and Turnover Are Disrupted in PE

FN matrix turnover is a dynamic balance between its synthesis, fibrillar assembly, and degradation (Wierzbicka-Patynowski and Schwarzbauer, 2003). In PE placentae and pMSCs, FN ER-to-Golgi transit is largely unaffected, while FN secretion and accumulation both within and outside the cells is increased. Also, DOC fractionation studies revealed that FN is primarily expressed and augmented in the insoluble protein fraction in both E-PE tissue and PE pMSCs relative to controls. This is in line with seminal studies conducted in human skin fibroblasts reporting that matrix-associated FN appeared as coarse insoluble fibrils, while cellular FN was primarily punctate or in short fibrils (McKeown-Longo and Mosher, 1983). Even under reducing conditions, we observed that insoluble FN in E-PE tissue and PE pMSCs consisted of large multimers greater than 480 in weight, while in controls, monomers of 245 were predominantly expressed. This suggests that the fibrils in PE pMSCs are likely immature and do not contribute to matrix integrity.

In support of published studies, we found that oxygen is a powerful mediator of FN protein levels in trophoblast cells (Caniggia et al., 1999; Genbacev et al., 2001; Chen and Aplin, 2003). Reflective of physiological changes in O_2_ in the placenta *in vivo*, FN levels are high in pMSCs from 6 to 9 weeks of gestation (when oxygen tension is low) and significantly drop at around 10–12 weeks (coincident with the opening of the intervillous space) remaining unchanged throughout pregnancy. This is in line with our *in vitro* data indicating an oxygen-dependent FN regulation in TC and PE pMSCs, which corroborates our previous observation of reduced oxygen availability leading to compromised JMJD6 activity in E-PE placental tissue (Alahari et al., 2018).

In addition to cleavage by ECM metalloproteinases (MMPs) (Shi and Sottile, 2011), a portion of FN undergoes lysosomal degradation (Sottile and Chandler, 2005). In line with this, we found that treatment of TC pMSCs with either lysosomal or autophagosome inhibitors led to intracellular FN accumulation and mimicked FN accumulation in PE pMSCs where FN was increasingly detected in the lysosomes. The buildup of insoluble, matrix-associated FN in PE (as evident from the DOC fractionation assay) shows that very little of this form is eventually endocytosed and recycled in this pathology. The importance of proper endocytic FN turnover for cell movement is exemplified by a study that reported that in migratory fibrosarcoma cells, once assembled FN fibrils are rapidly cleaved, internalized by endocytosis, degraded intracellularly by lysosomal proteases, and re-secreted *via* the late endosomal/lysosomal pathway to promote cell adhesion and motility (Humphries and Ayad, 1983; Sung and Weaver, 2011). This indicates a mechanism of directional cell migration that is contingent on FN proteolysis. Hence, it is plausible that lack of FN internalization and degradation in PE pMSCs directly impinge on their own FN migratory properties in pMSCs.

### Defects in ECM Deposition Underlie Impaired Extravillous Trophoblast Migration

In the present study, we employed primary pMSCs (Parveen, 2018) as a surrogate model of migratory, FN-producing cells that have undergone an epithelial–mesenchymal transition along the invasive pathway as EVTs (DaSilva-Arnold et al., 2018). These cells cross-talk with adjacent cytotrophoblast cells to influence their migratory and fusogenic ability (Talwadekar et al., 2015), through the secretion of soluble paracrine factors (Gerbaud et al., 2011). Given their fibroblast-like nature (Semenov et al., 2010), pMSCs are a significant source of FN (Chen et al., 2005; Mathew et al., 2017), thereby contributing to the formation of a structural and functional ECM. Similarly, fibroblast-secreted ECM has been shown to be critically involved in guiding directional cell migration and invasion in several *in vitro* and *in vivo* models (Chen and Aplin, 2003; Bonnans et al., 2014; Zeng et al., 2016; Erdogan et al., 2017). Importantly, we show here that healthy control pMSCs produce ECM that can support EVT migration *in vitro*. When matrix formation is pathological (as in the case of PE pMSCs, or upon overexpression of JMJD6), disorganization of fundamental components such as FN limit EVT migration. Our RGD peptide data support a key role for FN in modulating this event.

Emerging evidence implicates exosomes secreted during pregnancy, particularly those of placental origin, as powerful long-range influencers of cellular function by shuttling their cargo to target organs (Jin and Menon, 2018). Besides the secretion of paracrine factors, exosomes from pMSCs have been recently shown to directly affect HTR-8/SVneo migration and invasion by transporting factors such as specific miRNA (Chen et al., 2020). Herein, we observed that PE pMSC-derived exosomes contain more FN relative to controls; this has important implications for its interaction and signaling with other placental cells. Interestingly, we observed that not only do HTR-8/SVneo cells readily uptake pMSC-derived exosomes, but they also upregulated their cellular FN content upon exposure. Hence, it is plausible that exosomal FN uptake is partly responsible directly or indirectly for increased FN in HTR-8/SVneo cells, with the potential to influence their migratory and invasive capacity. Future studies are warranted to examine the precise mechanisms and consequences of PE pMSC-derived exosomes on EVT cell behavior.

### Correction of Cellular Iron Gradients by Hinokitiol Exposure Restored FN Deposition in PE

PE is characterized by profound alterations in placental iron homeostasis (Rayman et al., 2002). We recently reported that placental intracellular ferrous iron content is markedly reduced in PE, impinging on JMJD6 function (Alahari et al., 2018). Interestingly, despite compromised ferrous iron availability in the placenta, serum iron content is paradoxically elevated in PE primarily owing to increased hemolysis (Rayman et al., 2002). This apparent discrepancy may be explained by alterations in expression and regulation of ferroportin, the only known exporter of ferrous iron (Donovan et al., 2000) and enhanced activity and expression of the ferroxidase, ceruloplasmin (CP), with implications for impaired function of Fe^2+^-dependent enzymes (Shakour-Shahabi et al., 2010; Alahari et al., 2018). In the current study, we detected reduced Fe^2+^ content in pMSCs derived from PE pregnancies. One of the striking findings of the present study is the restoration of FN matrix deposition by PE pMSCs and, importantly, HTR-8/SVneo migration on the matrix deposited by the cells, upon exposure to Hinokitiol (4-isopropyltropolone), a natural compound extracted from the Taiwanese Hinoki tree with potent anti-oxidant and anti-tumor activities (Lee et al., 2013; Li et al., 2014). Highlighting its immense clinical potential, Hinokitiol was found to harness cellular iron gradients to selectively restore iron uptake in yeast cells and in mice lacking cellular iron transporters (Grillo et al., 2017). Its major clinical advantages include a strong affinity for binding to iron across lipid membranes, lack of cellular toxicity, or non-specific effects that are typically associated with iron chelators (Grillo et al., 2017). Using a fluorescent metalosensor assay, we report for the first time that addition of Hinokitiol to preeclamptic pMSCs significantly augmented the intracellular labile iron pool in these cells. This constitutes a reserve of transient, chelatable, non-protein-bound and accessible iron that is tightly maintained in a narrow range and can participate in key biochemical reactions (Kakhlon and Cabantchik, 2002). The depleted labile iron pool in PE pMSCs thus has ramifications for iron-dependent processes in these cells, including catalysis of JMJD6 enzyme activity and, as we report herein, FN turnover and deposition. As such, directly improving bioavailable iron in PE *via* Hinokitiol constitutes a critical point of intervention. Perhaps independently of its role in iron metabolism, Xu et al. (2017) reported that addition of Hinokitiol to human corneal epithelial cells enhanced their viability and protected against H_2_O_2_-induced oxidative stress, a well-established feature of preeclamptic cells. Collectively, these studies reiterate the tremendous clinical potential of this compound in reinstating overall cellular homeostasis.

## Conclusion

**FIGURE 8:**
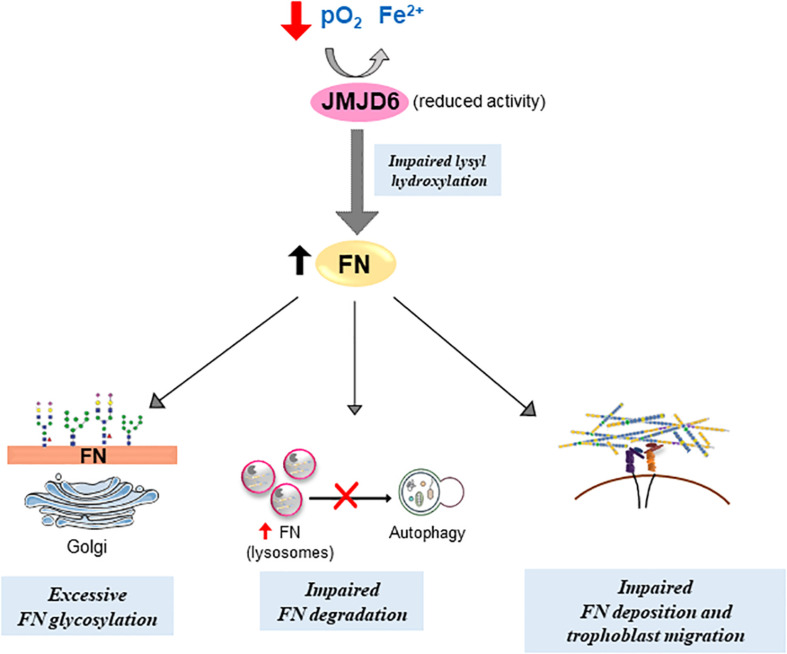
Putative model of impediments in FN matrix assembly and turnover in preeclampsia. Compromised JMJD6 enzyme activity due to hypoxia and reduced Fe^2+^ availability in PE placentae impedes proper FN glycosylation and deposition, resulting in its aberrant buildup. Concurrently, improper FN lysosomal degradation *via* autophagy further contributes to FN accumulation in PE. Altogether, defective FN processing diminishes trophoblast migration.

In conclusion, we describe a novel mechanism by which FN homeostasis and deposition is regulated by the lysyl hydroxylase JMJD6 in the human placenta. Reduced oxygen and iron availability, typical of pathological (preeclamptic) conditions, affect JMJD6 lysyl hydroxylase function, leading to alterations in FN processing and deposition (Figure 8), thereby affecting cell migration. The ability of Hinokitiol to restore the intracellular iron balance and JMJD6 function holds promise for targeting an understudied aspect of PE, namely, an altered iron balance and its impact on disrupted FN assembly and consequently cell migration.

## Data Availability Statement

The raw data supporting the conclusions of this article will be made available by the authors, without undue reservation.

## Ethics Statement

The studies involving human participants were reviewed and approved by the Mount Sinai Hospital Research Ethics Board (REB: 11-0287-E). The patients/participants provided their written informed consent to participate in this study.

## Author Contributions

SA: conceptualization, formal analysis, investigation, methodology, validation, visualization, and writing-original draft preparation. AF and LE: formal analysis, investigation, methodology, and validation. JS: formal analysis, investigation, and validation. CP, MR and ML: investigation and validation. TG: methodology and validation. MP: conceptualization, funding acquisition, methodology, writing—review and editing. IC: conceptualization, data curation, formal analysis, funding acquisition, methodology, project administration, resources, supervision, Writing—review and editing. All authors contributed to the article and approved the submitted version.

## Conflict of Interest

The authors declare that the research was conducted in the absence of any commercial or financial relationships that could be construed as a potential conflict of interest.
