# Supplementary material for: JMJD6 Dysfunction Due to Iron Deficiency in Preeclampsia Disrupts Fibronectin Homeostasis Resulting in Diminished Trophoblast Migration
Source: Front Cell Dev Biol. 2021 May 12;9:652607. doi: 10.3389/fcell.2021.652607 (PMC8149756; doi:10.3389/fcell.2021.652607)
Supplement: Supplementary file 7 [file Data_Sheet_1.PDF]

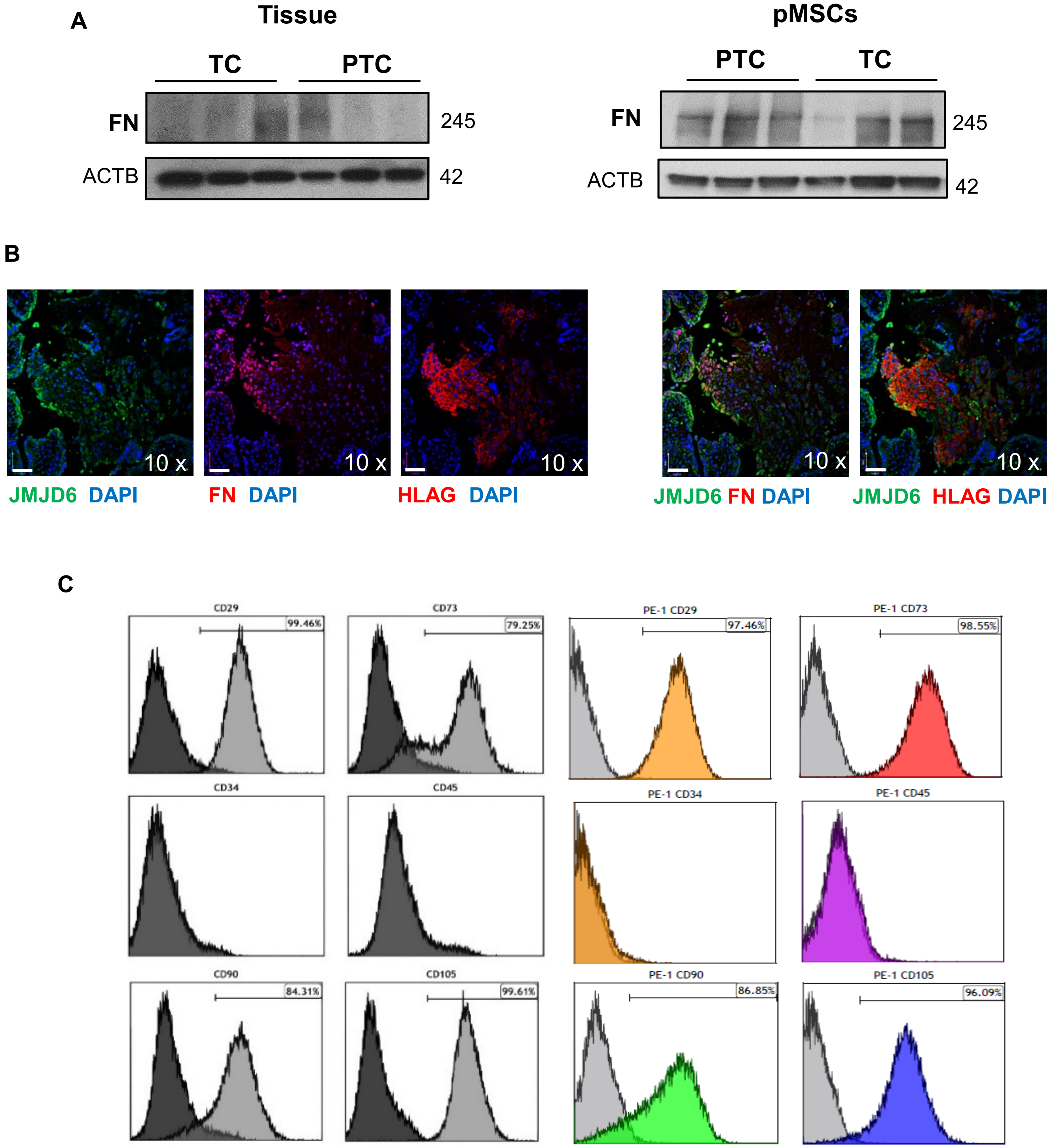

Supplementary Figure 1 – FN characterization in placental tissue and mesenchymal stromal cells (**A**) Representative Western blots of FN in pre-term control (PTC, n=9) *versus* term control (TC, n=8) placental tissue (left panel) and pMSCs (right panel). (**B**) IF analysis of JMJD6 (green), FN (red) and HLAG (red) in consecutive chorionic villus sections from a first trimester placenta (11 weeks gestation). Nuclei were counter-stained with DAPI. Scale bars at 10 X magnification represent 1.1 μM. (**C**) Fluorescent activated cell sorting (FACS) analyses of representative 7-week (left panels) and PE (right panels) pMSCs demonstrating the presence of mesenchymal stromal cell surface markers CD29, CD73, CD90 and CD105 and lack of hematopoietic markers, CD34 and CD45.

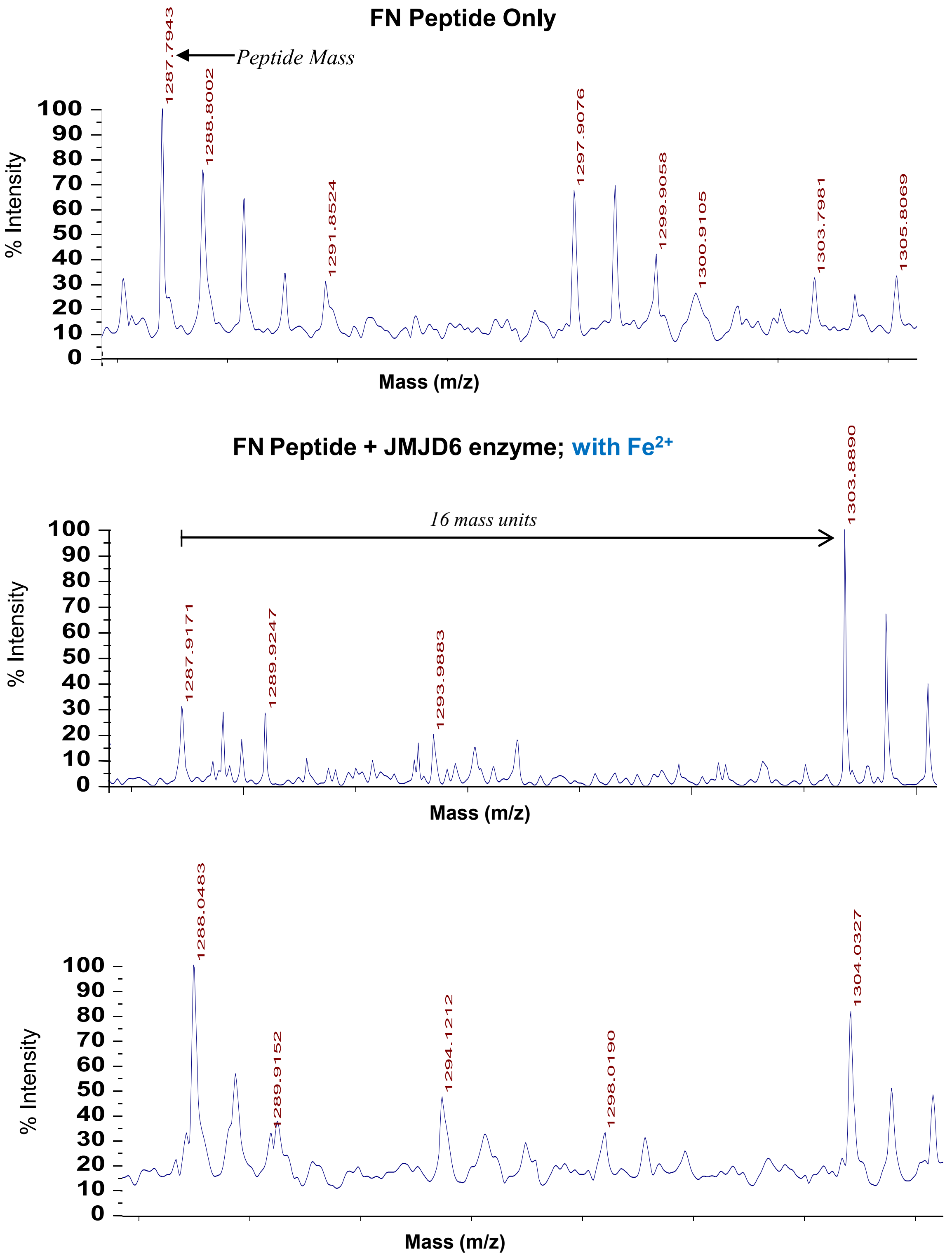

Supplementary Figure 2 – Iron is required for FN lysyl hydroxylation by JMJD6. Representative mass spectra for FN peptide showing the original peptide mass (top panel) and a 16-mass unit shift upon incubation with recombinant JMJD6 enzyme in the presence of ferrous iron (Fe^{2+}; middle panel) and to a lesser extent in the absence of Fe^{2+} (bottom panel).

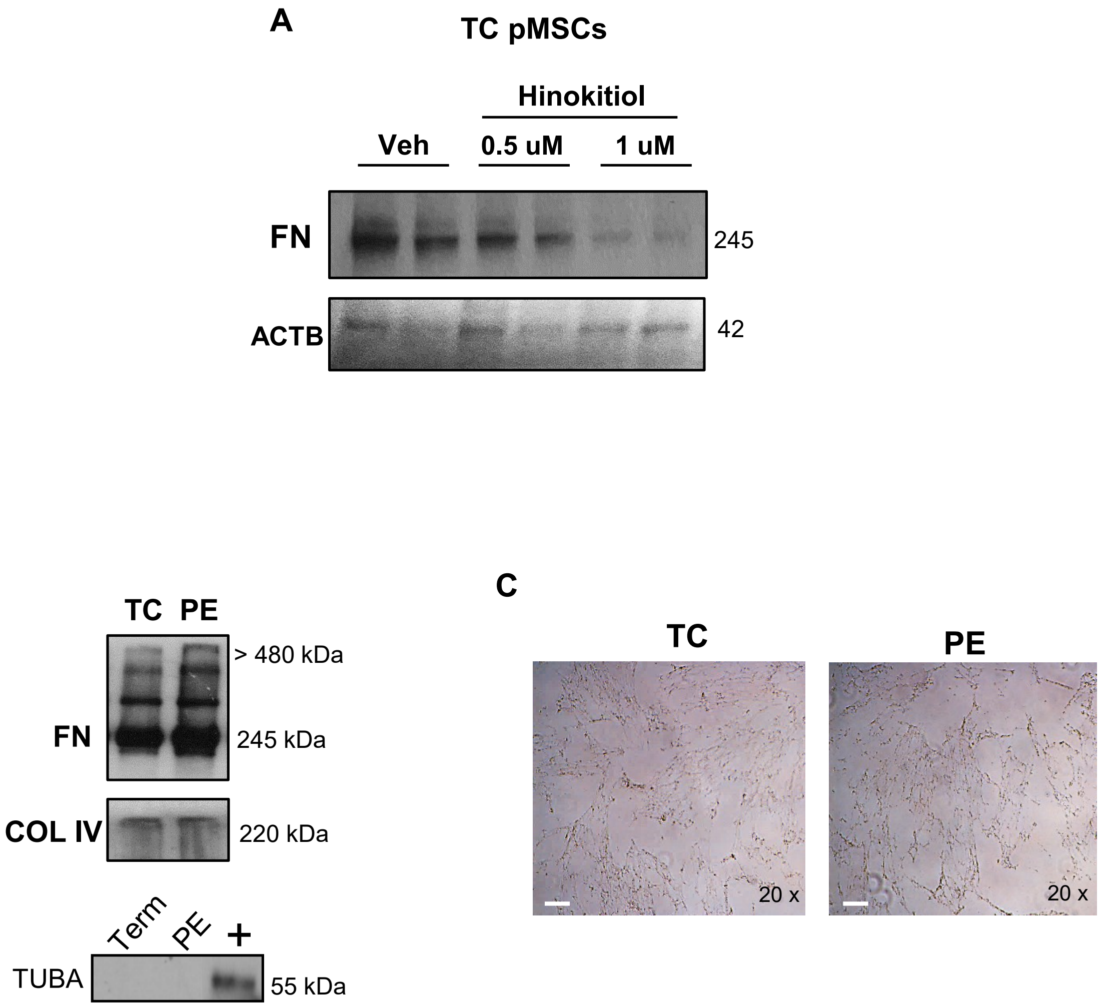

Supplementary Figure 3 – **Effect of Hinokitiol on control pMSCs and characterization of ECM isolated from pMSCs**. (**A**) WB for FN in Term pMSCs exposed to 0.5 or 1 μM Hinokitiol for 24 h. (**B**) WB for FN, Collagen IV (COL IV; (top panel) and α-tubulin (TUBA; bottom panel) in ECM isolated from TC and PE pMSCs. **+** = whole cell pMSC lysate as positive control for TUBA expression. (**C**) Representative phase contrast images of ECM isolated from TC and PE pMSCs. Scale bars represent 2.2 μM at 20 x magnification.

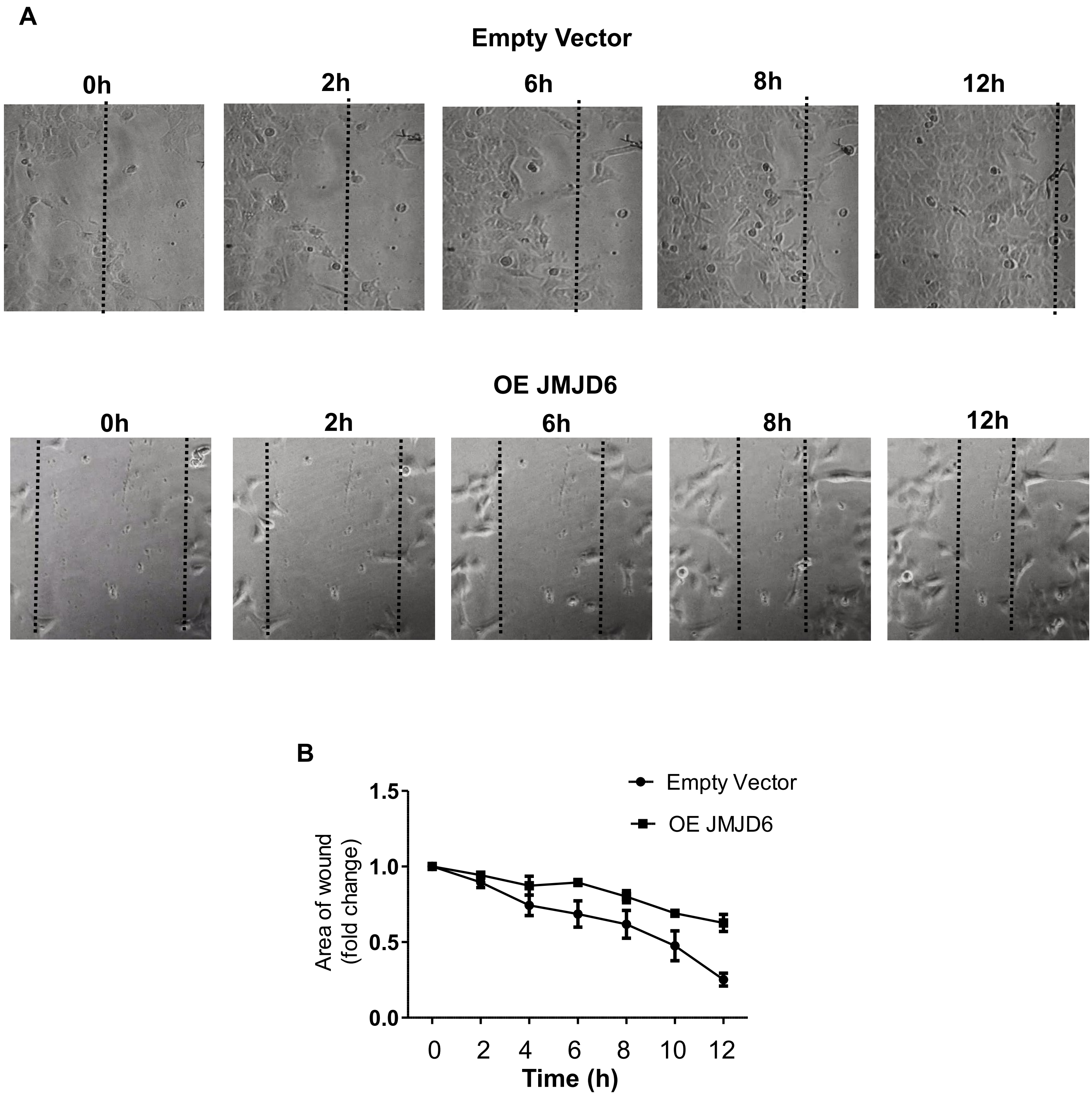

Supplementary Figure 4 – JMJD6 overexpression in pMSCs impedes HTR-8/SVneo migration *in vitro*. (**A**) Representative snapshots of HTR-8/SVneo cell migration over time on ECM deposited by TC pMSCs transfected with empty vector control or 0.5 µg JMJD6 vector. (**B**) Graphical representation of HTR-8/SVneo cell migration on TC pMSC-derived ECM following JMJD6 OE, expressed as area of wound closure over time.

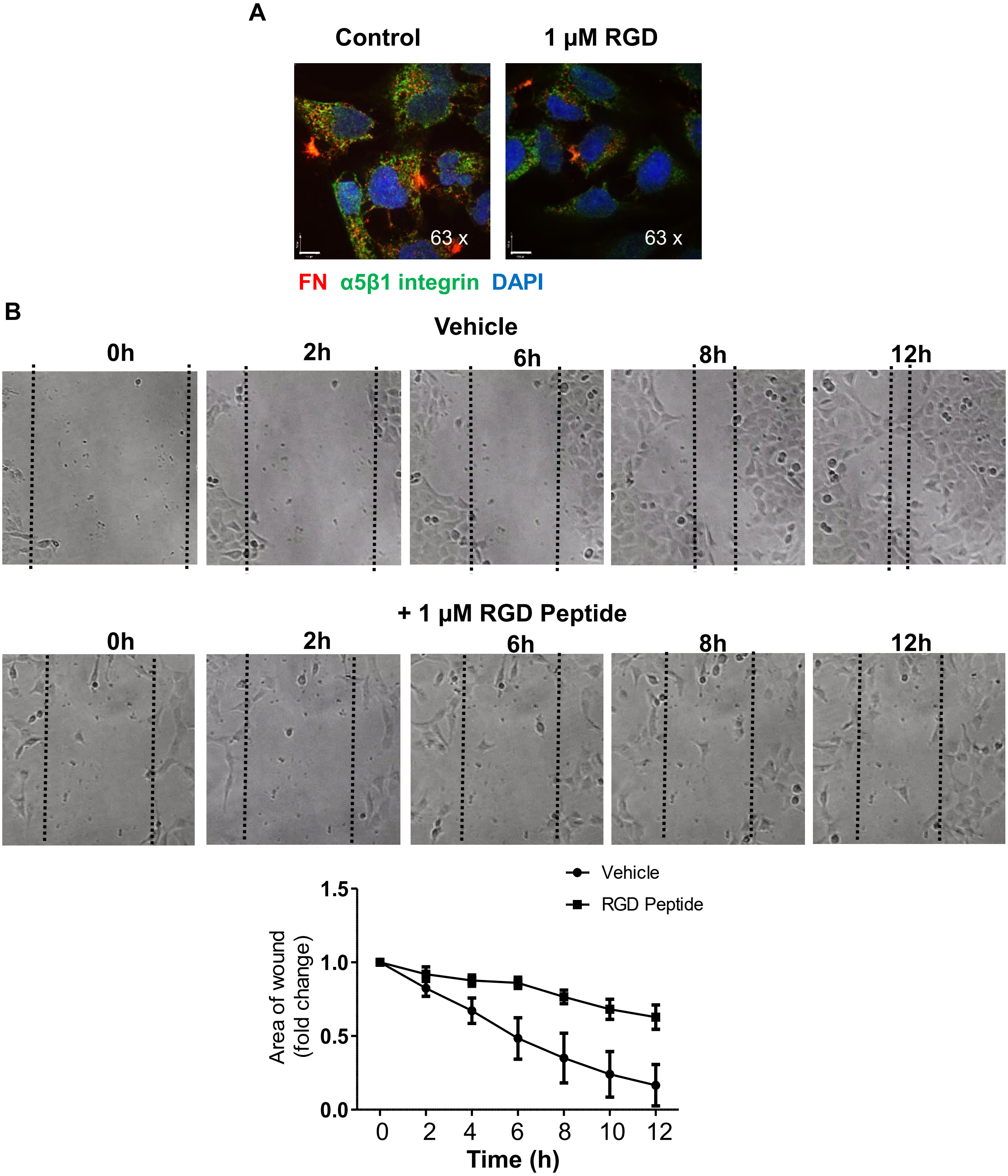

Supplementary Figure 5 – Inhibition of FN ligand-receptor interaction in TC pMSC-derived ECM diminished HTR-8/Svneo cell migration (**A**) IF analysis of FN (red) and α5β1 integrin (green) in HTR-8/Svneo cells plated on term pMSC-derived ECM exposed to 1μM RGD peptide for 90 min. Scale bars at 63 X magnification represent 7 μM. (**B – top panel**) Representative snapshots of HTR-8/Svneo cell migration over time on ECM deposited by TC pMSCs exposed to 1μM RGD peptide. (**B – bottom panel**) Graphical representation of HTR-8/SVneo cell migration on pMSC ECM exposed to 1μM RGD peptide, expressed as area of wound closure over time.

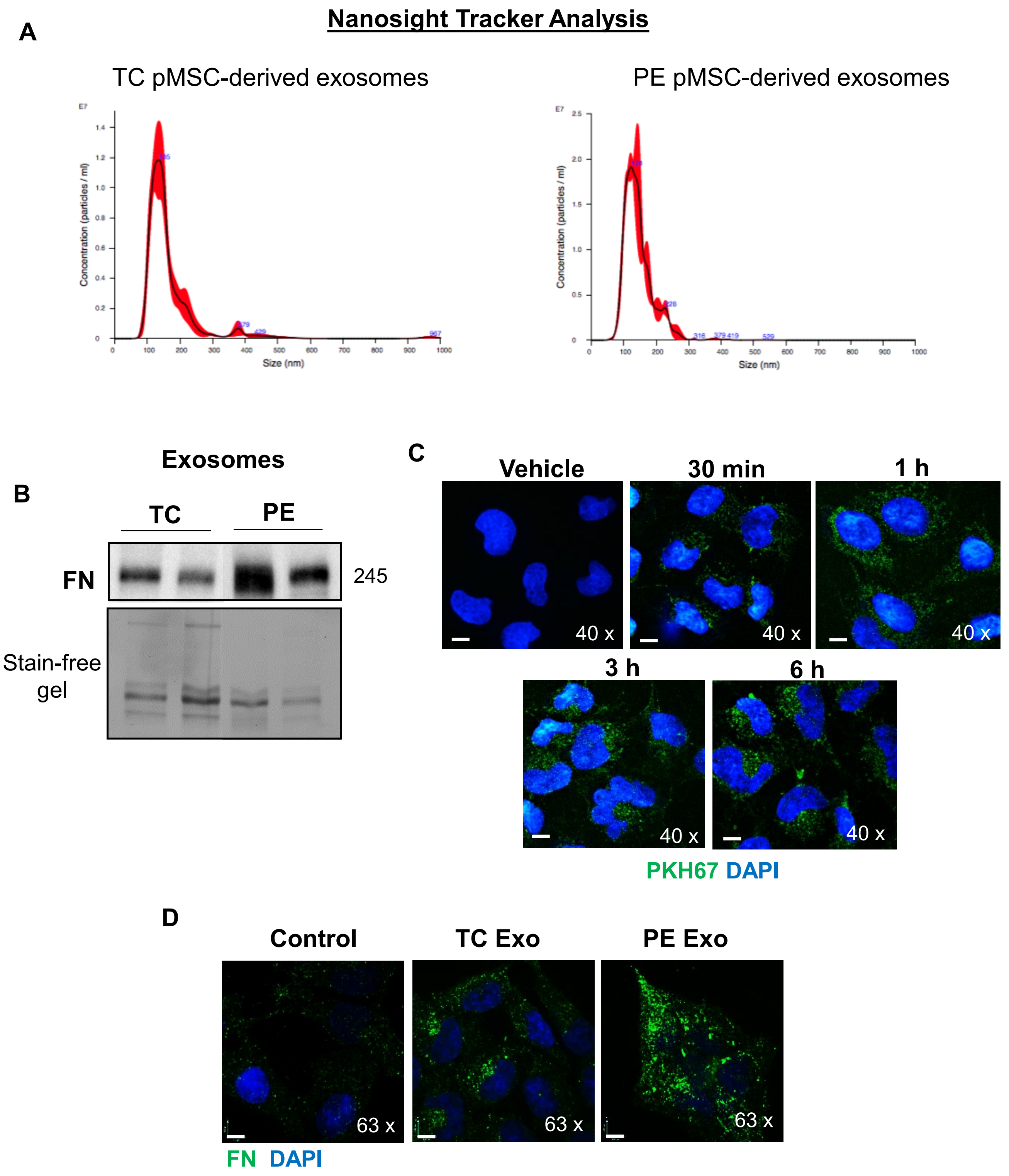

Supplementary Figure 6 – Fibronectin expression in pMSC-derived exosomes, their uptake and effect on HTR-8/Svneo cells. (**A**) Representative Nanosight tracker analysis of TC and PE-derived exosomes isolated from conditioned media. (B) WB for FN in TC and PE pMSC-derived exosomes. (**C**) IF analysis of PKH67 linker dye (green) marking TC pMSC-derived exosomes uptaken by HTR-8/Svneo cells over time. Nuclei were counter-stained with DAPI. (**D**) IF analysis of FN (green) in HTR-8/Svneo cells exposed to exosomes isolated from TC *versus* PE pMSCs. Nuclei were counter-stained with DAPI. Scale bars at 40 X and 63 X magnification represent 4.44 and 7 μM respectively.
